# Medical Education e-Professionalism (MEeP) framework; from conception to development

**DOI:** 10.1080/10872981.2021.1983926

**Published:** 2021-11-13

**Authors:** Shaista Salman Guraya, Salman Y. Guraya, Denis W. Harkin, Áine Ryan, Mohd Zarawi bin Mat Nor, Muhamad Saiful Bahri Yusoff

**Affiliations:** a Royal College of Surgeons Ireland, Adliya, Kingdom of Bahrain; b Department of Medical Education, School of Medical Sciences, Universiti Sains Malaysia, Kubang Kerian, Malaysia; c Clinical Sciences Department, College of Medicine, University of Sharjah, Sharjah, UAE; d Faculty of Medicine and Health Sciences, Royal College of Surgeons in Ireland, Dublin, Ireland

**Keywords:** e-professionalism, digital professionalism, professionalism, framework, social networking sites

## Abstract

**Background:**

Medical professionalism education intends to produce virtuous and humanistic healthcare professionals who demonstrate perseverance and professional integrity. However, today’s medicine has embodied a mammoth transformation of medical practice towards sns and the digital realm. Such paradigm shift has challenged the medical professional’s values, behaviors, and identities, and the distinct boundaries between personal and professional lives are blurred. This study aims to develop a framework for healthcare professionals coping with the challenges of medical professionalism in the digital realm.

**Methods:**

We followed a systematic approach for the development of a framework about e-professionalism. Qualitative data was collected from a systematic review and a delphi study, while quantitative data was collected by administering a validated questionnaire social networking sites for medical education (snsme). Subsequently, categorization of the selected data and identifying concepts, deconstruction and further categorizing concepts (philosophical triangulation), integration of concepts (theoretical triangulation), and synthesis and resynthesis of concepts were performed.

**Results:**

The initial process yielded six overlapping concepts from personal, professional, character (implicit) and characteristic (explicit) domains: environment, behavior, competence, virtues, identity, and mission. Further integration of data was done for the development of the medical education e-professionalism (meep) framework with a central concept of a commitment to mission. The mission showed deep connections with values (conformity, beneficence, universalism, and integrity), behaviours (communication, self-awareness, tolerance, power), and identity (reflection, conscientiousness, self-directed, self-actualization). The data demonstrated that all medical professionals require updated expertise in sns participation.

**Conclusion:**

The meep framework recognises a mission-based social contract by the medical community. This mission is largely driven by professional values, behaviors and identity. Adherence to digital standards, accountability, empathy, sensitivity, and commitment to society are essential elements of the meep framework.

## Introduction

Medical professionalism is a multifaceted construct that encompasses the expected virtues, behaviours and attitudes of healthcare professionals. Electronic, or e-professionalism entails the attitudes and behaviors described through traditional professionalism strands, that are manifested through SNS [1]. Similarly, digital professionalism, may be considered an interchangeable term with e-professionalism, referring to ‘the use of traditional concepts of medical professionalism while drawing attention to the particular opportunities and challenges afforded by professionals’ use of digital media and how the profession is changed by this use’ [2]. Use of Social Networking Sites (SNS) has expanded, and this phenomenon has catalyzed both the speed and scale of our interactions [3]. Amplified by the power of the Internet and Internet-based technologies, enormous volumes of information and content can be accessed instantly, by individuals and allows interaction with other users. Today’s users of SNS often value speed-of-access to information, rather than accuracy [4]. A plethora of digital interfaces such as vlogs, microblogs, folksonomies, wikis, and other Internet-based applications are being used for marketing, social, and educational purposes [5]. As it stands, the use of SNS has significantly expanded among healthcare professionals, but the education and training of e-professionalism have not advanced reciprocally [6].

In the medical discipline, healthcare professionals use SNS for networking, education, marketing, promotion, patient care and awareness campaigns [7,8]. Healthcare professionals use SNS such as Twitter, Facebook, Instagram, LinkedIn and WeChat to secure professional relations and communication with patients and their families [9–12]. SNS provide a powerful means for information exchange, immediate access to healthcare information, and even emotional and psychological consultancy [13]. Another outright benefit of SNS comes from its usage for medical research for collecting patient-data, understanding perceptions, and assessing public impact [14,15]. SNS may also provide platforms for medical communities of practice to interact both professionally and socially and provide and receive training and education. However, its usage amongst the medical community becomes sensitive on account of its characteristics of being ‘powerful’, ‘permanent’, and ‘public’ especially as the third party rights of patients are at stake [16].

There are concerns by healthcare professionals that the expanding usage of SNS in the medical field is not without hazards. Despite securing privacy settings, the borderless horizons of the digital world can be accessed by infinite clients in one way or another [17]. Unfortunately, boundaries between professional and personal lives become blurred, and lapses in professional behaviour can result, which can leave both a permanent and very visible digital imprint [18]. A proliferation of reports, have highlighted online violations of professional ethics by medical students, faculty, and healthcare professionals [19]. An example is excessive self-disclosure by healthcare professionals on SNSs, which is considered an utter violation of professional conduct [20]. A large body of literature has reported the illegal posting of patients’ photos and personal data by healthcare professionals, often without explicit consent, which can adversely affect patient–doctor relationships [21]. The American Medical Association has warned that the ubiquitous use of SNS by healthcare professionals has resulted in an information ecosystem that contains true and false information, with the potential to confuse or misinform patients and the public [21]. Such ‘Fake news’, disinformation, or poor-quality health information can undermine patients’ confidence in healthcare professionals and the profession. Furthermore, unprofessional acts witnessed on SNS by healthcare professionals have included venting, profanity, discriminatory language, and inappropriate remarks about patients [22]. Clearly, these should not be tolerated in either the physical or virtual world.

Although there is a staggering rise of SNS usage among healthcare professionals, patients, and their families, little is known of the impact this has had on privacy, confidentiality, and other important aspects of our professional codes of conduct. The concept of e-professionalism is still in its’ infancy in the medical field, and policies for the use of SNS amongst medical professionals are considered primitive and rudimentary [23]. In order to address the ethical conduct of healthcare professionals, three separate frameworks have been published which focus on different constructs of medical professionalism. First, the virtue-based professionalism framework focuses on moral character and reasoning with an emphasis on a set of values steered by moral reasoning [24]. Second, the behaviour-based professionalism framework relies on professional milestones and competencies, which appreciates correct behaviors and applies sanctions to unprofessional behaviors [25,26]. Third, the professional identity formation (PIF) based framework recognizes the importance of positive role models and has the ability to alert healthcare professionals about the occurrence of negative role models [27]. However, these three diverse approaches have not yet been unified into a single coherent framework for medical professionals that describes the expected professional virtues, behaviors and identity in respect to e-Professionalism. Furthermore, the rapidly evolving landscape of SNS, and digital communication, brings repeated challenge to embrace new methods of communication, representation and conduct. In this context, there is no current framework of medical professionalism that can address the new discourse of e-professionalism.

This study aims to develop a Medical Education e-Professionalism (MEeP) framework which can describe healthcare professionals’ expected conduct using SNSs. This will help us to understand the complexity of e-professionalism, the issues related to the blurring of personal and professional boundaries and arising from liberal information sharing. We have attempted to incorporate professional values, behaviours, and identity from the digital realm in a single framework that complement, and help unify, three separate construct-based professionalism frameworks.

## Study design

We designed our study strategy by using the research philosophy of Bilau et al., which uses three fundamental steps for conducting a framework-developing investigation [28]. This approach included; *ontology*, the perception of being subjective or objective in the real world; *epistemology*, the realm of understanding from reflections; and *axiology*, the researchers’ persona of opinions and beliefs. Integrating these three legacies, we performed a deductive (rigorous evaluation of several propositions emerging from theory) as well as inductive (analysis of a given phenomenon by recognition of themes and trends) approaches in our study [29].

We conducted this study by performing a systematic review for an in-depth analysis of the published literature, a survey-based investigation using a validated inventory Social Networking Sites for Medical Education (SNSME) [30,31], and performing a Delphi study for capturing the experts’ opinions. Table 1 outlines our research philosophy and paradigms, including research questions for developing the Medical Education e-Professionalism (MEeP) framework.

**Table 1.** Research philosophy and paradigms

Research questions	Ontology	Epistemology	Axiology
To gather information and data, related to suggested e-professionalism.	Knowledge – existing social phenomena. Idealism was applied	Qualitative literature review – interpretative approach	Value – laden
What is the degree, nature (social or educational) and professional use of SNS?	Knowledge – existing social phenomena. Idealism was applied	Reality – a result of human mind, data from stakeholders’ opinion – Interpretivist approach	Value – laden
What are the desired values and behaviors of digital professionalism that are needed for maintaining digital professional identity?	Knowledge – outside the social phenomena. Realism applied	Experts’ opinions – Pragmatist’s approach	Value – free

## Systematic Review [32]

We used Preferred Reporting Items for Systematic Reviews and Meta-analyses (PRISMA) guidelines for data mining and selection of the studies for this systematic review [33]. Our research questions were based on Sample, Phenomenon of Interest, Design, Evaluation and Research type (SPIDER) [34]. We looked into the published literature for the desired values and behaviors for a digital professional identity formation and the impact of SNS in an educational setting. On 11 May 2020, we performed a search on PubMed, ProQuest, ScienceDirect, Web of Science, and EBSCO host using keywords (professionalism AND (professionalism OR (professional identity) OR (professional behaviors) OR (professional values) OR (professional ethics) AND (SNS) AND (SNS) OR (social networking sites) OR Twitter® OR Facebook® OR Instagram® OR WeChat®) AND (healthcare professionals) for the English-language articles published between 1 January 2015 and 30 April 2020 (**Appendix 1**). We defined our sample ‘healthcare professionals’ as individuals engaged in the delivery of the healthcare system, including healthcare professionals, nurses, dentists, physiotherapists, and pharmacists. The phenomenon of interest was defined as the digital world including SNS, social networking sites, Twitter®, Facebook® and Instagram®, and WeChat®. The original research articles with qualitative, quantitative and mixed methods studies about digital professionalism, e-professionalism in the digital age, guidelines for the usage of SNS, and the degree and extent of usage of SNS by healthcare professionals for educational, professional and personal purposes were included. We excluded systematic reviews, scoping reviews, meta-analysis, editorials, and commentaries from our search. While the thematic analysis was used for emerging concepts and theories, the leading themes and concepts were further analyzed in discussion to reach consensus for future implementation. For the analysis itself, the SSG and SYG coded all articles and constructed a coding tree. Later on, other investigators critically analyzed and selected final themes about digital professionalism.

## Survey-based research

The SNSME questionnaire was distributed to all undergraduate medical students at the Royal College of Surgeons Ireland – Medical University of Bahrain (RCSI-MUB) through SurveyMonkey®. The medical curriculum at RCSI-MUB is delivered in a six-year programme, comprising Foundation Year till year 5. The curriculum is the same as the one delivered in RCSI Dublin [35]. The students are from 38 nationalities giving a very diverse and multicultural aspect to the student cohort. The SNSME inventory contains 29 questions about the frequency and type of personal electronic communication for the general, educational and professional use of SNS (e.g., Facebook, YouTube, Twitter, LinkedIn, WeChat, and Flickr) on a 5-point Likert agreement scale **(Appendix 2)**. There was one open-ended question about the participants’ opinions about e-professionalism in SNS. The data analysis was done using the Statistical Package for Social Sciences (SPSS) v.20. As all statements were arranged on an ordinal scale, inferential statistics were performed by non-parametric tests. As a prerequisite to using other non-parametric tests, the normality of data was verified by a one-sample Kolmogorov-Smirnov test. The Mann-Whitney U test was used to compare the differences between genders, while the Kruskal Wallis test was applied to compare the differences between more than two independent groups. A p-value <0.05 was considered significant.

## Delphi technique

Delphi technique collects anonymous opinions from a panel of experts to forecast future trends on a given research topic [36]. The process starts with Delphi questions which focus on identifying problems and tangible solutions. The research statements and concerns for the subsequent rounds are generated from the responses of preceding rounds. The process comes to an end on receiving answers to the research question. In our research, a representative group of 36 experts on SNS usage, professionalism and e-professionalism traits from different regions, nationalities, ethnicity and culture were invited to contribute to the Delphi study on e-professionalism. The expert panel was recruited by using the maximum variation sampling technique [37]. Participants were required to respond across all three rounds to complete the Delphi process. A dropout rate of 20% was expected over the three rounds. Upon agreement, round 1 questionnaire was administered via SurveyMonkey®, which asked for single words or short statements (descriptors) to be submitted against each of the following three questions:

1. Who is the Man (Person) of character in the digital world?

2. Who is the Man (Person) of characteristics in the digital world?

3. How is professional identity acquired in the digital world?

During round 2, the descriptors were grouped into categories which were then classified into themes [38]. All descriptors, categories, and themes were distributed to the expert panel to rank from strongly disagree to strongly agree. There was no option for free-text responses in round 2. The returned rankings of the expert panel were manually analyzed. In round 3, each participant received an individualized survey comprising all categories, which were presented alongside the participants’ own responses and the group’s responses (percentage agreement/disagreement) from round 2. Participants were asked to reconsider their responses in the light of the group’s responses for a final review and opinion. A consensus on essential values and behaviours leading to PIF in the digital world was sought.

To develop the framework using the data from the three studies, we followed a structured process using a grounded theory approach, which suggested a continuous interplay between data and analysis [39,40]. During this process the results were identified as being in agreement (convergence), offering complementary information on the same topic (complementarity), or appearance of information contradicting each other (discrepancy or dissonance) [41].

- Mapping the selected data sources
- Extensive reading, categorization of the selected data, and identification of concepts
- Mapping of concepts across studies **–** philosophical triangulation
- Integration of concepts – theoretical triangulation
- Synthesis, resynthesis, and making it all make sense – Development of the MEeP framework

## Ensuring research rigor

We followed the four standard steps by Lincoln and Guba 1994 [42] for ensuring research rigor; credibility, transferability, dependability, and conformability. These included administration of relevant questions with a detailed context (credibility), theoretical underpinnings of our findings that can be generalized by other researchers for their studies (transferability). We integrated methods and results (dependability) and reduced researchers’ bias by an iterative approach of data analysis during different stages of the research to ensure that results were not unduly swayed (conformability).

## Ethical approval

Ethical approval for this study was granted by the University Sains Malaysia (USM/JEPeM/19050291) and by the Royal College of Surgeons Ireland Medical University Bahrain (RCSI-MUB/REC/15032020). All participants provided informed consent to participate at the beginning of the online survey and in the Delphi process. All data were handled in accordance with the European General Data Protection Regulation [43] as well as all relevant regulations and Data Protection legislation in Bahrain.

## Results

### Mapping the selected data sources

**Figure 1.**
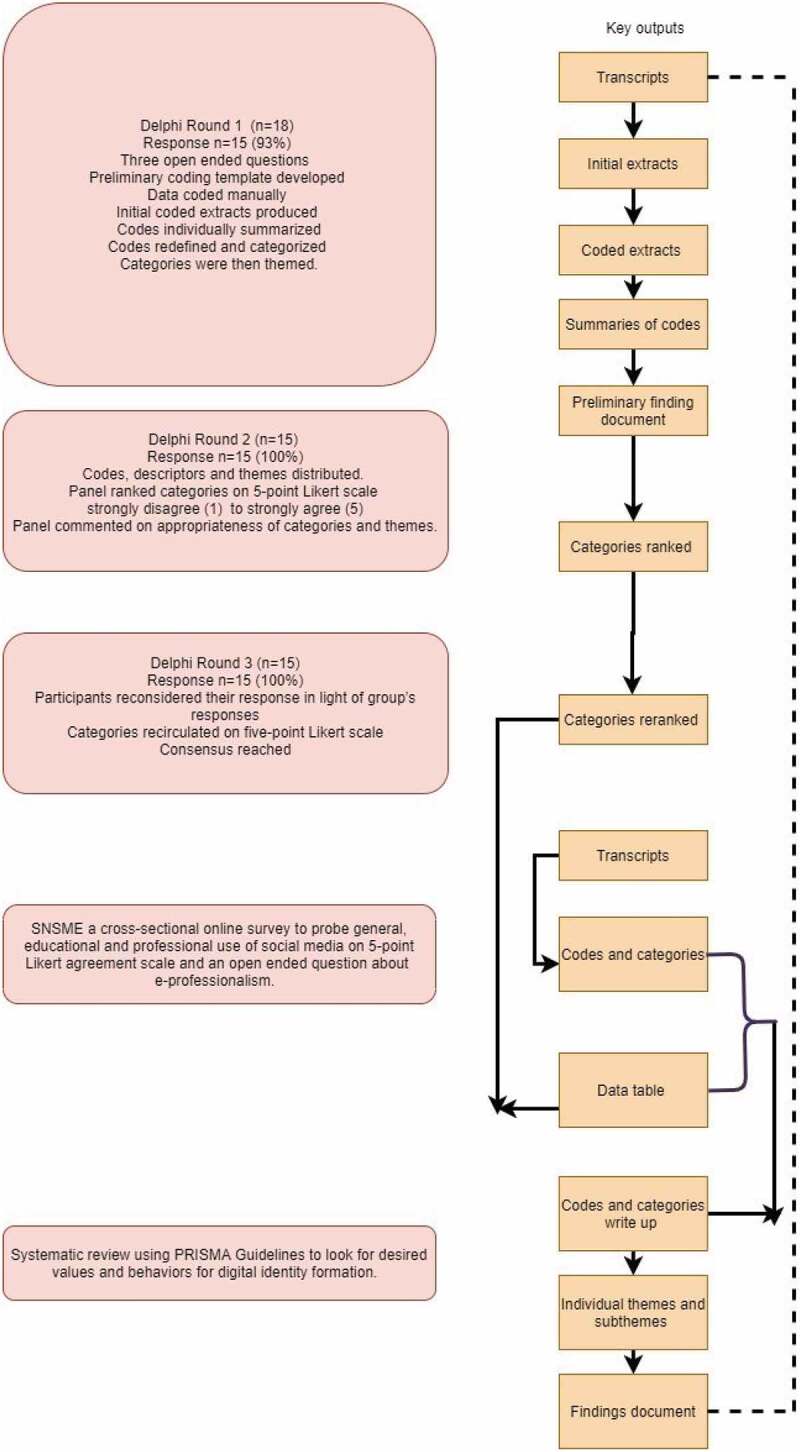
A structured algorithm of methodological triangulation of scoping review, SNSME survey and Delphi study

*A mapping of the collected evidence* was carried out by triangulation of the retrieved data from a methodological perspective. (Figure 1)

SNSME; Social Networking Sites in Medical EducationPRISMA; Preferred Reporting Items for Systematic Reviews and Meta-analyses

*The systematic review* initially yielded 4055 references. Further analysis shortlisted 44 articles for further analysis. Most studies were based in the USA (15/34%), while other studies were based in Canada (8/18%), UK (4/9%), China (3/7%), UAE (3/7%), and New Zealand (1/3%). The staged analysis of the selected studies generated four leading threads; (1) an escalating rise in the use of SNS by healthcare professionals and students, (2) a negative impact of SNS on digital professionalism, (3) blurring of medical professional values, behaviors, and identity in the digital era, and (4) a shred of limited evidence for teaching and assessing digital professionalism. A high occurrence of a violation of patient privacy and professional integrity and cyberbullying was observed.

Of 977 invitees, 381 [244 (64%) female and 137 (36%) male] responded to the *SNSME survey* (a response rate of 39%). The majority of respondents 213 (56%) were in the age group 18–20 years, 122 (32%) students were in 21–23 years, and 46 (12%) students were older than 23 years. The data showed that there were 59 (15.4%) students from foundation year, 75 (19.6%) students from 1^st^ year, 49 (12.9%) students from 2^nd^ year, 74 (19.5%) students from 3^rd^ year, 64 (16.9%) students from 4^th^ year, and 60 (15.7%) 5^th^ year students. The majority (105; 27.6%) students used email once a week to share educational material, while 223 (58.6%) students used SNS to remain in touch with their peers and tutors. Interestingly, 88 (23%) students used SNS for sharing education-related information 3 to 5 times a day. As many as 109/381 (28.7%) students did not get any formal instructions for the use of SNS by their institution, while only 118/381 (31%) were aware of a formal SNS policy. Generally, though the use of SNS for leisure and fun was high, the students were not aware of its code of conduct and legislative complexities (**Appendix 3 and Appendix 4**). Using the Mann-Whitney U test for the comparison of genders’ responses, showed a significant variation in two statements (*p* < 0.05). The responses of male students were significantly in favour of the statement ‘*social networking sites help me access educational resources*’ with a high mean rank of 147.13 compared to 121.39 mean rank for female students. In contrast, female students’ responses significantly favoured the statement *‘I have posted content (images/videos/text) on SNS that could be considered unprofessional’* than the male counterparts, mean ranks of 140.69 and 113.20, respectively. Comparing the students’ responses across years of schooling, the results of Kruskal Wallis test showed significant differences for two statements for educational use of SNS, and one statement for professional use of SNS, respectively (*p* < 0.05). The most significant difference was noted for the statement *‘social networking sites are useful in developing reading and writing web skills*’, where senior students agreed much more than the junior students. The responses to the open-ended question of the SNSME inventory highlighted the importance of being honest, responsible, and possess good communication and interpersonal skills. ‘User ratings’ and the complexity of acquiring an appropriate digital identity were prominent.

In the *Delphi study*, of the 36 invited experts, 18 participants completed round 1 (response rate 50%), 15 of 18 completed round 2 (response rate 83.3%), and 15 of 15 completed round 3 (response rate 100%). Approximately 118 descriptors were analyzed and were subsumed into 39 categories which generated 13 themes **(Appendix 5)**. The number of categories with *strongly agree* consensus improved significantly for each theme from rounds 2 to 3. In round 2, 100% consensus was achieved for 20/39 (51.2%) categories, which rose to 100% for all categories in round 3. The identified themes were categorised as follows.

- Conformity: self-restraint and subordination of one’s own inclinations to the expectations of others
- Benevolence: preserve and enhance welfare of those with whom one is in frequent personal contact
- Power: status and prestige, control people and resources
- Self-direction: autonomous thought and action (idea of agency)
- Universalism: tolerance and concern for welfare of all others
- Achievement: competitive personal success
- Honest
- Responsible
- Self-aware
- Reflective
- Conscientious
- Altruistic
- Communicator

## Extensive reading, categorization of the selected data, and identification of concepts

We applied a bottom-up approach, and the discrete findings were linked with each other, thus classifying patterns and identifying unique concepts [44]. We analyzed the data from systematic review, SNSME survey, and Delphi study, where recurring descriptors and categories were generated carrying common links with themes by SSG and SYG. These descriptors, categories and themes were further cross verified and validated by other four authors DWH, AR, MZN and MSBY. An excerpt, of quotations from different studies is presented in a tabular format as **Appendix 6**

**Figure 2.**
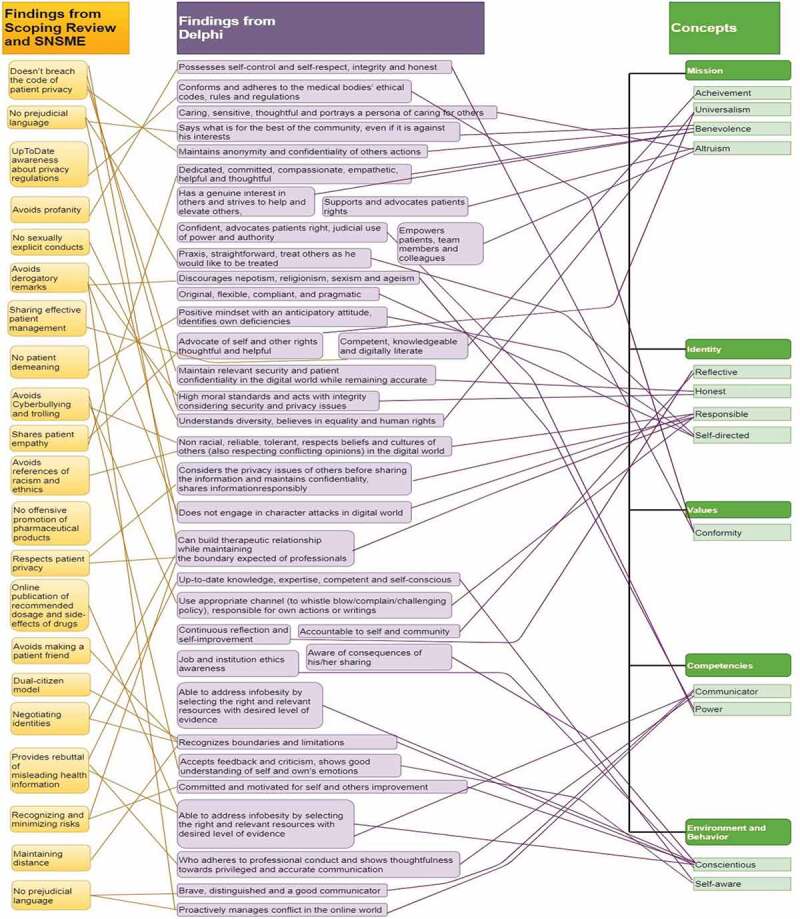
A structured layout of the phased emergence of concepts of e-professionalism and their integration towards the MEeP framework

During this data analysis process, the bespoke relevance of categories and themes emerged as concepts; *digital environment, behaviours, competencies, values, identity*, and *mission*. Interestingly, though not intended purposefully, the concepts of our research analysis fit closely with Korthagen’s model of change [45]. A comprehensive demonstration of the phased emergence of concepts and their integration towards the conceptual analysis is shown in Figure 2.

We considered the identified concepts. The *digital environment* was considered as fluid, diverse, autonomous, and connected. The *Behaviors*, as the correct conduct in the digital environment, which in turn is heavily influenced by *Competencies*, which constitute the knowledge, skills, and attitudes which determine the potential for correct conduct. *Values and beliefs* reflect the degree of conscious awareness of one’s abilities and knowledge of digital world. *Identity* pertains to personal and professional characteristics, values, and norms; the ‘who am I: personal and professional self’. Finally, *Mission*, signifies the driving force that fosters a specific role of self, or ‘status’.

## Mapping of concepts across studies – philosophical triangulation

**Figure 3.**
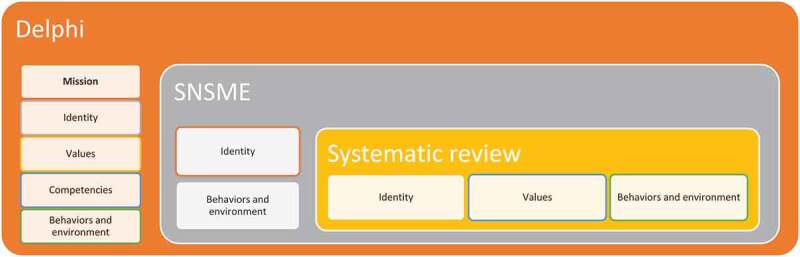
The expected outcomes from a medical professional in the digital era resulting from the triangulation of data from scoping review, SNSME survey and Delphi study

Data from systematic review and SNSME deduced idealism, interpretive, and value-laden characteristics of a healthcare professional in the digital world. In contrast, *the data* from Delphi study valued realism, pragmatism and value-free traits of e-professionalism in the medical field. The concept mapping from systematic review, SNSME survey, and Delphi study highlighted the overlapping similarities except for mission that emerged from Delphi study only. The outcome of triangulation from each study is shown in Figure 3.

SNSME, Social Networking Sites for Medical Education

## Integration of concepts – theoretical triangulation

At this stage, we integrated the collected concepts with theoretical perspectives. This exercise showed six concepts from personal and professional paradigm. Values, identity and mission represent implicit aspects, while competencies and behaviours in environment are more explicit expressions. Individuals relate to each other in different social roles; hence the concept of personal and professional identity emerges. At the same time, a continuous presentation of ‘Professional self’ can undermine personal identity formation, which was not the case in the pre-internet world. Henceforth there is a need for a single identity in the digital world as there are no personal and professional boundaries. That digital identity can be achieved if one has the driving force and commitment that fosters a specific role of ‘self’.

## Synthesis, resynthesis, and making it all make sense – Development of framework

**Figure 4.**
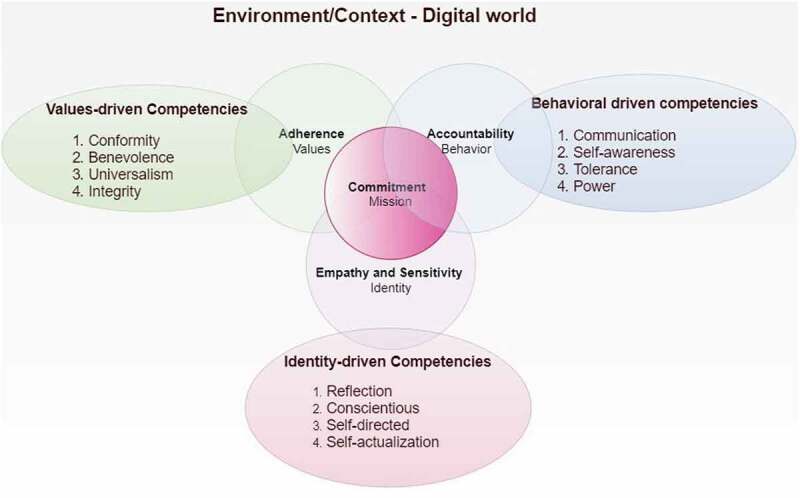
The Medical Education e-professionalism (MEeP) framework

Following an iterative process of synthesis and resynthesis, the researchers reached a consensus to develop the MEeP framework (Figure 4).

The iterative nature of data analysis from these different lenses emphasized, the complexities of the digital world, and that mere alignment of all these concepts could not attain the mission. Instead the three essential concepts; of values, behavior and identity need to complement each other and converge on a cynosure; a ‘Mission.’ That Mission, a commitment to the social contract of a medical professional with society, intertwining professional values (adherence to standards), behaviors (accountability) and identity (empathy and sensitivity). To achieve that mission, a specific set of competencies are required from all three levels.

## Discussion

This study represents a structured approach to the development of a unifying Medical Education e-Professionalism (MEeP) Framework for the digital realm. The mainstay of the framework is the ‘Mission’, – a commitment by the medical profession to society through a social contract to deliver healthcare services with appropriate professional values, behaviors and identity. The MEeP framework addresses the medical mission, reflecting a list of competencies (Figure 4) that can be used by academics for curricular reforms and by learners for their own self-assessment and reflection. In the pursuit of this mission, in the digital-era healthcare professionals should preserve and embody professional values, behaviors, and identities. Medical professionalism incorporates a code of healthcare ethics and constitutes the execution of professional virtues in practice. Virtues which demonstrate an intermix of values, attitudes, and behaviors to promote a patient-centered and compassionate care, delivered through perseverance and with integrity [24]. Correspondingly, our framework has revealed a special symbiotic relationship between our commitment to mission and adherence to standards (values), accountability (behaviors), and empathy and sensitivity (identity). This mission based MEeP framework is further interconnected with competencies from values, behaviors, and identity that when embraced may assist healthcare professionals as they carefully navigate their conduct through the digital world.

Medical professionalism has a fundamental ethos of morality, which underpins the need to understand *the Man (Person) of character* and *the Man (Person) of characteristics* who is deciding or executing crucial actions in the digital realm. In this digital environment, the attention capacities, memory process, and social cognition have profoundly deteriorated [46]. Continuous presence in the digital environment has been shown to exhaust cognitive reserves and undermine neuro-cognitive outcomes leading to impaired impulse-control and decision-making [47]. These mechanisms predict the social behaviors of Internet users and may also potentially influence behaviors of healthcare workers on SNS. While developing a narrative-based framework for medical professionalism, Dr Jack Coulehan has asserted the need for a technical professional identity [48]. If we accept this narrative, tech-savvy and digitally expert medical professionals would be preferred in the modern world as they may be better equipped to recognize personal and professional territories without endangering patient’s integrity, privacy, and social norms [49]. Similarly, self-awareness and narrative competence are considered to be essential facets in fostering healthcare workers’ fitness to practice [50]. Our framework brings up the role of a medical professional by being mindful of the expected professional values, behaviors, and identities. The MEeP framework advocates that being *a Man (Person) of character*, a healthcare worker would acquire competencies of professional values (conformity, benevolence, universalism, integrity) while interacting with colleagues, patients and their families, and community stakeholders. On the other hand, being a *Man (Person) of characteristics*, a healthcare professional is accountable for communication, self-awareness, tolerance and self-restrained in the digital world.

An essential construct of our MEeP mission-based framework is based on professional behaviors in the digital realm. Healthcare professionals’ behaviors in the digital environment may not predict the healthcare professionals’ behaviors in the physical environment; they may be professionally sound in the hospitals while they may behave unprofessionally on SNS. An example is the online sharing of a patient’s picture or a part of the body without formal consent. According to the theory of connectivism, SNS usage is impulsive, uninhibited, and immersive [51,52]. There is a thin line between personal and professional landscapes in SNS, and that’s where infringements of professional behaviors can occur. The ubiquity of the Internet access and its’ numerous intersections with diverse professional activities such as communication, consultations, awareness, and accountability mean digital professionalism should be folded into modern medical competencies and entrustable professional activities [2]. There is a pressing need to embed a thread of core principles of e-professionalism into the fabric of all medical curriculum, for undergraduate, postgraduate and continued professional education.

We have viewed the main concepts from both personal and professional paradigms. When examined through the optics of identity in the digital era, Holden at al., have argued that a gap exists in the conduct of a virtuous person between his (their) physical and online space [53]. Knight and Mattick, have eluded that the epistemological perceptions of medical students and healthcare workers gradually evolve by experience, incremental self-awareness, and self-identities with a resultant PIF [54]. It follows therefore, since such experiences are not consistent, that the various incongruous events individual may encounter will result in ‘identity patching’ with deficits in some perhaps crucial areas. With further experience, medical professionals tend to secure ‘identity splinting’ where their PIF supports their patched identity to minimise those deficits. These phenomena become crucial in the digital world as we experience a context collapse, which denotes a widening aperture between physical and cyberspace [55]. Context collapse takes place when heterogeneous groups, content, and moral values from diverse settings converge through SNSs [56]. Henceforth, an understanding of the environment (contextualized work) plays a vital role in mitigating the risk of collapsed contexts. Otherwise, this phenomenon can flatten versatile audiences into a common group with a weak situation-specific dimension of PIF. There is a wealth of research about the blurring of contextual boundaries while using SNSs by medical professionals [3,31,57]. This highlights how the personal and professional aspects of one’s life can be managed in the co-existence of physical space. However, once the environment is devoid of this buffering effect, the pervasive context collapse blurs this separation in the digital world. Although numerous strategies to offset this contextual collapse are used, such as self-disclosures, self-censorship, audience-specific digital adaptations, and privacy settings, there remain situations where online presence is inappropriate in terms of values, competencies, behaviors, and identities. The between and betwixt; a liminal approach [58] cannot work in the digital realm and demands an early convergence and alignment of multiple identities to one ‘Self’ to ‘Status’ [59]. Where status may equate to our ‘Mission’ – a conception of self-existence. That’s how the concept of ‘Mission’ emerged in our study, which remains unchanged irrespective of the digital or physical environment.

Analysing mission from a different perspective, the Hippocratic oath eloquently testifies that, carrying a noble mission, a medical graduate will embrace universal ethics, beneficence, and non-maleficence [60,61]. However, in the digital world, preserving professional identity and self-presentation during interactions on SNS has shown precarious instances of how healthcare professionals construe their conduct [62]. Beneficence might have been preserved in the physical world, but, unknowingly, a physician might have posted a patient’s details on SNSs with an infringement of codes of confidentiality and privacy. This brings up the value of the MEeP framework to revisit the professional identities while adhering to the core mission of medicine [63–65]. As evident from the literature, SNS has transformed the medical field into a web-based avenue for PIF and socialization [66]. There is a need to cultivate a presentation of self that is devoid of personal and professional aspects, but at the same time meet the demands of both.

The aforementioned concepts with theoretical and philosophical triangulations have helped us to develop the mission based MEeP framework, which focuses on singularity rather than a plurality of the individual. For healthcare professionals, the core tenets of e-professionalism, including reflections, caring, conscientiousness, accountability, and commitment to duty, should be mastered along with a sound knowledge of SNS. Alignment of such sets of competencies with the digital environment, which is impulsive, intercultural, and powerful, will diversify the scope of medical professionals. Currently, there is a scarcity of instructions and guidelines in medical curricula about the professional use of SNS in the digital realm. Our study demands formal training and evaluation of the medical trainees and professionals to prepare them for their future digitally enhanced medical practice.

## Considerations for Implementation

Due to the complex nature of teaching behavioural skills, more novice learners can be educated about the professional mission and identity (status and self) components of the framework by introducing exercises which focus on self-reflection and self-actualization. Once the learners grasp the main concepts of the medical profession’s mission and their own professional identities, the value-driven components of the MEeP framework (e.g., conformity, integrity and benevolence) can be introduced into the curriculum. Vertical integration of MEeP framework components is recommended and will reinforce concepts for learners.

## Study limitations

This study used a diverse range of research methodologies to triangulate and analyse data. The research design is a kind of mixed methods protocol where both quantitative and qualitative approaches were adopted. Despite a thorough protocol, the chances of eliminating research bias cannot be guaranteed. More evidence-based multi-centre studies on this novel phenomenon would be advantageous. We didn’t explicitly used TikTok in our search strategy.

## Conclusion

This study has developed a novel and unifying mission-based Medical Education e-Professionalism (MEeP) Framework, which focuses on the core principles of professionalism and the duties of our social contract. Recognizing that SNS, due to online disinhibition and both reach and permanency of digital footprint, are challenging environments for the maintenance of appropriate professional values, behaviors, and identities. Henceforth, we propose that our MEeP framework may guide the acquisition of necessary competencies of e-Professionalism and enable us to fulfill our commitments to society through the creation of an appropriate ‘one own self’ in the digital environment. This framework can be conveniently adopted by medical institutions and healthcare authorities to guide healthcare professionals in the digital realm for the acquisitions of desired attributes.

## Figures and Tables

**Figure:**
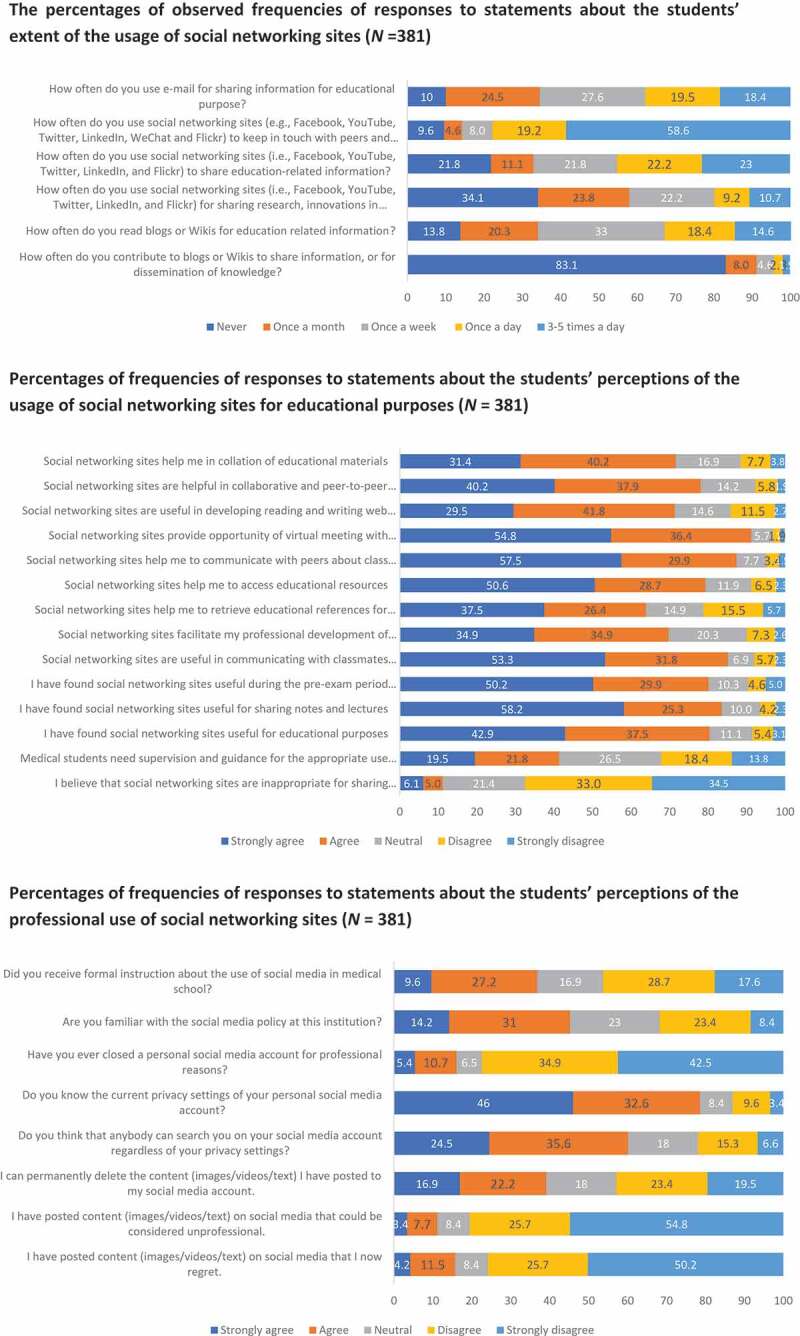
The percentages of observed frequencies of responses to statements about the students’ extent of the usage of social networking sites (*N* = 381)

## Appendix 1.Search strategy – PUBMED 12 May 2020 (n = 1388)

Search: **(((((((Professionalism) OR (‘professionalism’[MeSH Terms] OR ‘professionalism/education’[MeSH Terms] OR ‘professionalism/ethics’[MeSH Terms] OR ‘professionalism/trends’[MeSH Terms] OR ‘professionalism/standards’[MeSH Terms])) OR (professional attributes)) OR (professional values)) OR (professional behaviors)) OR (professional identity)) AND ((((SNS) OR (‘SNS’[MeSH Terms] OR ‘SNS/ethics’[MeSH Terms] OR ‘SNS/standards’[MeSH Terms] OR ‘social networking’[MeSH Terms] OR ‘social networking/ethics’[MeSH Terms])) OR (Twitter)) OR (facebook))) AND (healthcare professionals)** Filters: **Journal Article, from 2015–2020**

((((((((((((((((‘professional’[All Fields] OR ‘professional s’[All Fields]) OR ‘professionalism’[MeSH Terms]) OR ‘professionalism’[All Fields]) OR ‘professionality’[All Fields]) OR ‘professionalization’[All Fields]) OR ‘professionalize’[All Fields]) OR ‘professionalized’[All Fields]) OR ‘professionalizing’[All Fields]) OR ‘professionally’[All Fields]) OR ‘professionals’[All Fields]) OR ((((‘professionalism’[MeSH Terms] OR ‘professionalism/education’[MeSH Terms]) OR ‘professionalism/ethics’[MeSH Terms]) OR ‘professionalism/trends’[MeSH Terms]) OR ‘professionalism/standards’[MeSH Terms])) OR (((((((((((‘professional’[All Fields] OR ‘professional s’[All Fields]) OR ‘professionalism’[MeSH Terms]) OR ‘professionalism’[All Fields]) OR ‘professionality’[All Fields]) OR ‘professionalization’[All Fields]) OR ‘professionalize’[All Fields]) OR ‘professionalized’[All Fields]) OR ‘professionalizing’[All Fields]) OR ‘professionally’[All Fields]) OR ‘professionals’[All Fields]) AND ((((((((‘attributable’[All Fields] OR ‘attribute’[All Fields]) OR ‘attribute s’[All Fields]) OR ‘attributed’[All Fields]) OR ‘attributes’[All Fields]) OR ‘attributing’[All Fields]) OR ‘attribution’[All Fields]) OR ‘attributional’[All Fields]) OR ‘attributions’[All Fields]))) OR (((((((((((‘professional’[All Fields] OR ‘professional s’[All Fields]) OR ‘professionalism’[MeSH Terms]) OR ‘professionalism’[All Fields]) OR ‘professionality’[All Fields]) OR ‘professionalization’[All Fields]) OR ‘professionalize’[All Fields]) OR ‘professionalized’[All Fields]) OR ‘professionalizing’[All Fields]) OR ‘professionally’[All Fields]) OR ‘professionals’[All Fields]) AND (‘value’[All Fields] OR ‘values’[All Fields]))) OR (((((((((((‘professional’[All Fields] OR ‘professional s’[All Fields]) OR ‘professionalism’[MeSH Terms]) OR ‘professionalism’[All Fields]) OR ‘professionality’[All Fields]) OR ‘professionalization’[All Fields]) OR ‘professionalize’[All Fields]) OR ‘professionalized’[All Fields]) OR ‘professionalizing’[All Fields]) OR ‘professionally’[All Fields]) OR ‘professionals’[All Fields]) AND (((((((((((((((((‘behavior’[MeSH Terms] OR ‘behavior’[All Fields]) OR ‘behavioral’[All Fields]) OR ‘behavioural’[All Fields]) OR ‘behavior s’[All Fields]) OR ‘behaviorally’[All Fields]) OR ‘behaviour’[All Fields]) OR ‘behaviourally’[All Fields]) OR ‘behaviours’[All Fields]) OR ‘behaviors’[All Fields]) OR ‘pattern’[All Fields]) OR ‘pattern s’[All Fields]) OR ‘patternability’[All Fields]) OR ‘patternable’[All Fields]) OR ‘patterned’[All Fields]) OR ‘patterning’[All Fields]) OR ‘patternings’[All Fields]) OR ‘patterns’[All Fields]))) OR (((((((((((‘professional’[All Fields] OR ‘professional s’[All Fields]) OR ‘professionalism’[MeSH Terms]) OR ‘professionalism’[All Fields]) OR ‘professionality’[All Fields]) OR ‘professionalization’[All Fields]) OR ‘professionalize’[All Fields]) OR ‘professionalized’[All Fields]) OR ‘professionalizing’[All Fields]) OR ‘professionally’[All Fields]) OR ‘professionals’[All Fields]) AND (‘identities’[All Fields] OR ‘identity’[All Fields]))) AND (((((‘SNS’[MeSH Terms] OR (‘social’[All Fields] AND ‘media’[All Fields])) OR ‘SNS’[All Fields]) OR ((((‘SNS’[MeSH Terms] OR ‘SNS/ethics’[MeSH Terms]) OR ‘SNS/standards’[MeSH Terms]) OR ‘social networking’[MeSH Terms]) OR ‘social networking/ethics’[MeSH Terms])) OR ((‘twitter’[All Fields] OR ‘twitter s’[All Fields]) OR ‘twitters’[All Fields])) OR (“facebook “[All Fields]))) AND ((((‘health personnel’[MeSH Terms] OR (‘health’[All Fields] AND ‘personnel’[All Fields])) OR ‘health personnel’[All Fields]) OR (‘health’[All Fields] AND ‘professionals’[All Fields])) OR ‘healthcare professionals’[All Fields])

**Translations**

**Professionalism**: ”professional”[All Fields] OR ‘professional’s’[All Fields] OR ‘professionalism’[MeSH Terms] OR ‘professionalism’[All Fields] OR ‘professionality’[All Fields] OR ‘professionalization’[All Fields] OR ‘professionalize’[All Fields] OR ‘professionalized’[All Fields] OR ‘professionalizing’[All Fields] OR ‘professionally’[All Fields] OR ‘professionals’[All Fields]

**professional**: ”professional”[All Fields] OR ‘professional’s’[All Fields] OR ‘professionalism’[MeSH Terms] OR ‘professionalism’[All Fields] OR ‘professionality’[All Fields] OR ‘professionalization’[All Fields] OR ‘professionalize’[All Fields] OR ‘professionalized’[All Fields] OR ‘professionalizing’[All Fields] OR ‘professionally’[All Fields] OR ‘professionals’[All Fields]

**attributes**: ”attributable”[All Fields] OR ‘attribute’[All Fields] OR ‘attribute’s’[All Fields] OR ‘attributed’[All Fields] OR ‘attributes’[All Fields] OR ‘attributing’[All Fields] OR ‘attribution’[All Fields] OR ‘attributional’[All Fields] OR ‘attributions’[All Fields]

**professional**: ”professional”[All Fields] OR ‘professional’s’[All Fields] OR ‘professionalism’[MeSH Terms] OR ‘professionalism’[All Fields] OR ‘professionality’[All Fields] OR ‘professionalization’[All Fields] OR ‘professionalize’[All Fields] OR ‘professionalized’[All Fields] OR ‘professionalizing’[All Fields] OR ‘professionally’[All Fields] OR ‘professionals’[All Fields]

**values**: ”value”[All Fields] OR ‘values’[All Fields]

**professional**: ”professional”[All Fields] OR ‘professional’s’[All Fields] OR ‘professionalism’[MeSH Terms] OR ‘professionalism’[All Fields] OR ‘professionality’[All Fields] OR ‘professionalization’[All Fields] OR ‘professionalize’[All Fields] OR ‘professionalized’[All Fields] OR ‘professionalizing’[All Fields] OR ‘professionally’[All Fields] OR ‘professionals’[All Fields]

**behaviors**: ”behavior”[MeSH Terms] OR ‘behavior’[All Fields] OR ‘behavioral’[All Fields] OR ‘behavioural’[All Fields] OR ‘behavior’s’[All Fields] OR ‘behaviorally’[All Fields] OR ‘behaviour’[All Fields] OR ‘behaviourally’[All Fields] OR ‘behaviours’[All Fields] OR ‘behaviors’[All Fields] OR ‘pattern’[All Fields] OR ‘pattern’s’[All Fields] OR ‘patternability’[All Fields] OR ‘patternable’[All Fields] OR ‘patterned’[All Fields] OR ‘patterning’[All Fields] OR ‘patternings’[All Fields] OR ‘patterns’[All Fields]

**professional**: ”professional”[All Fields] OR ‘professional’s’[All Fields] OR ‘professionalism’[MeSH Terms] OR ‘professionalism’[All Fields] OR ‘professionality’[All Fields] OR ‘professionalization’[All Fields] OR ‘professionalize’[All Fields] OR ‘professionalized’[All Fields] OR ‘professionalizing’[All Fields] OR ‘professionally’[All Fields] OR ‘professionals’[All Fields]

**identity**: ”identities”[All Fields] OR ‘identity’[All Fields]

**SNS**: ”SNS”[MeSH Terms] OR (‘social’[All Fields] AND ‘media’[All Fields]) OR ‘SNS’[All Fields]

**Twitter**: ”twitter”[All Fields] OR ‘twitter’s’[All Fields] OR ‘twitters’[All Fields]

**facebook**: ”facebook”[All Fields] OR ‘facebook’s’[All Fields]

**healthcare professionals**: ”health personnel”[MeSH Terms] OR (‘health’[All Fields] AND ‘personnel’[All Fields]) OR ‘health personnel’[All Fields] OR (‘health’[All Fields] AND ‘professionals’[All Fields]) OR ‘healthcare professionals’[All Fields]

**ProQuest 13 May 2020 (n = 1348)**

**Table:**

#	Searches	Results
S1	Professionalism	**209,963**
S2	Professional identity	**851,518**
S3	Professional behaviours	**1,586,079**
S4	Professional values	1,902,786
S5	Professional ethics	**722,966**
S6	SNS	**1,820,584**
S7	Social networking sites	**164,903**
S8	Twitter	**332,511**
S9	Facebook	**255,642**
S10	Healthcare professionals	2,722,521
S11	professionalism OR (professional identity) OR (professional behaviors) OR (professional values) OR (professional ethics)	**2,394,519**
S12	professionalism AND (professionalism OR (professional identity) OR (professional behaviors) OR (professional values) OR (professional ethics))	**209,959**
S13	(SNS) OR (social networking sites) OR Twitter OR facebook	**2,160,035**
S14	(SNS) AND ((SNS) OR (social networking sites) OR Twitter OR facebook)	**2,160,035**
S15	(professionalism AND (professionalism OR (professional identity) OR (professional behaviors) OR (professional values) OR (professional ethics))) AND ((SNS) AND ((SNS) OR (social networking sites) OR Twitter OR facebook)) AND (healthcare professionals) -	**91,368**
S16	(professionalism AND (professionalism OR (professional identity) OR (professional behaviors) OR (professional values) OR (professional ethics))) AND ((SNS) AND ((SNS) OR (social networking sites) OR Twitter OR facebook)) AND (healthcare professionals) -	4448

Databases:

Ebook Central

Health Research Premium Collection

ProQuest Dissertations & Theses Global

Narrowed by:

Entered date: 2015–05-13 – 2020-05-13;Subject: higher education; nursing; health education; medicine; students; medical personnel; health care; pedagogy; professionals; nurses; behavioral psychology; health sciences; hospitals; questionnaires; medical research; instructional design; medical education; attitudes; medical ethics;Language: English

**ISI Web of Science (n = 50)**

**Table:**

#	Searches	Results
#1	TOPIC: (professionalism) *Indexes = SCI-EXPANDED, CPCI-S Timespan = All years*	6,813
#2	TOPIC: (professional identity) *Indexes = SCI-EXPANDED, CPCI-S Timespan = All years*	4,092
#3	TOPIC: (professional behaviours) *Indexes = SCI-EXPANDED, CPCI-S Timespan = All years*	20,587
#4	TOPIC: (professional ethics) *Indexes = SCI-EXPANDED, CPCI-S Timespan = All years*	7,191
#5	TOPIC: (professional values) *Indexes = SCI-EXPANDED, CPCI-S Timespan = All years*	20,955
#6	TOPIC: (SNS) *Indexes = SCI-EXPANDED, CPCI-S Timespan = All years*	38,891
#7	TOPIC: (social networking sites) *Indexes = SCI-EXPANDED, CPCI-S Timespan = All years*	6,477
#8	TOPIC: (Twitter) *Indexes = SCI-EXPANDED, CPCI-S Timespan = All years*	12,217
#9	TOPIC: (Facebook) *Indexes = SCI-EXPANDED, CPCI-S Timespan = All years*	9,119
#10	TOPIC: (healthcare professionals) *Indexes = SCI-EXPANDED, CPCI-S Timespan = All years*	102,885
#11	#5 OR #4 OR #3 OR #2 OR #1 *Indexes = SCI-EXPANDED, CPCI-S Timespan = All years*	53,046
#12	#11 AND #1 *Indexes = SCI-EXPANDED, CPCI-S Timespan = All years*	6,813
#13	#9 OR #8 OR #7 OR #6 *Indexes = SCI-EXPANDED, CPCI-S Timespan = All years*	52,490
#14	#13 AND #6 *Indexes = SCI-EXPANDED, CPCI-S Timespan = All years*	38,891
#15	#14 AND #12 AND #10 *Indexes = SCI-EXPANDED, CPCI-S Timespan = All years*	66
#16	#14 AND #12 AND #10 Refined by: PUBLICATION YEARS: (2020 OR 2017 OR 2019 OR 2016 OR 2018 OR 2015) *Indexes = SCI-EXPANDED, CPCI-S Timespan = All years*	50

**Science direct/Scopus (n = 1230)**

Medical professionalism AND SNS

**EBSCO host (2015-2020) 13 May 2020 (n = 39)**

Professionalism AND SNS AND Healthcare professionals

## Appendix 2 . Social Networking Sites in Medical Education (SNSME)

**Age (in years)**

**Country of residence:**

**Is country of residence same as nationality**: Yes No

**(If no, please specify) -**

**Gender** Male Female Prefer not to disclose

**Year in MBBS course** FY 1 2 3 4 5 6

**Table:**

No.	Question	Never	Once a month	Once a week	Once a day	3–5 times a day
1	How often do you use e-mail for sharing information for educational purpose?					
2	How often do you use social networking sites (e.g., Facebook, YouTube, Twitter, LinkedIn, WeChat and Flickr) to keep in touch with peers and tutors?					
3	How often do you use social networking sites (i.e., Facebook, YouTube, Twitter, LinkedIn, and Flickr) to share education-related information?					
4	How often do you use social networking sites (i.e., Facebook, YouTube, Twitter, LinkedIn, and Flickr) for sharing research, innovations in medicine, and updates in medical field?					
5	How often do you read blogs or Wikis for education related information?					
6	How often do you contribute to blogs or Wikis to share information, or for dissemination of knowledge?

**Table:**

No.	Question	Strongly agree	Agree	Neutral	Disagree	Strongly disagree
7	Social networking sites help me in collation of educational materials					
8	Social networking sites are helpful in collaborative and peer-to-peer learning					
9	Social networking sites are useful in developing reading and writing web skills					
10	Social networking sites provide opportunity of virtual meeting with other students and tutors					
11	Social networking sites help me to communicate with peers about class projects					
12	Social networking sites help me to access educational resources					
13	Social networking sites help me to retrieve educational references for research					
14	Social networking sites facilitate my professional development of learning skills in technology					
15	Social networking sites are useful in communicating with classmates about course-related topics					
16	I have found social networking sites useful during the pre-exam period when I get an instant answer/explanation from my peer, instead of going through the books					
17	I have found social networking sites useful for sharing notes and lectures					
18	I have found social networking sites useful for educational purposes					
19	Medical students need supervision and guidance for the appropriate use of social networking sites for educational purposes					
20	I believe that social networking sites are inappropriate for sharing classroom materials, information, and discussing healthcare related topics					
21	Did you receive formal instruction about the use of SNS in medical school?					
22	Are you familiar with the SNS policy at this institution?					
23	Have you ever closed a personal SNS account for professional reasons?					
24	Do you know the current privacy settings of your personal SNS account?					
25	Do you think that anybody can search you on your SNS account regardless of your privacy settings?					
26	I can permanently delete the content (images/videos/text) I have posted to my SNS account.					
27	I have posted content (images/videos/text) on SNS that could be considered unprofessional.					
28	I have posted content (images/videos/text) on SNS that I now regret.					

29. Please write any other ideas and opinions about e-professionalism in SNS that you consider to be important.

## Appendix 3. Pictorial presentations of SNSME Results

**Percentages of frequencies of responses to statements about the students’ perceptions of the usage of social networking sites for educational purposes (*N* = 381)**

**Percentages of frequencies of responses to statements about the students’ perceptions of the professional use of social networking sites (*N* = 381)**

## Appendix 4. Tabular presentations of SNSME Results

**The percentages of observed frequencies of responses to statements about the students’ extent of the usage of social networking sites (*N* = 381)**

**Table:**

	Male (n = 137)					Female (n = 244)				
	Never	Once a month	Once a week	Once a day	3–5 times a day	Never	Once a month	Once a week	**Once a day**	**3–5 times a day**
How often do you use e-mail for sharing information for educational purpose?	12	32.6	21.7	19.6	14.1	8.9	20.1	30.8	19.5	**20.7**
How often do you use social networking sites (e.g., Facebook, YouTube, Twitter, LinkedIn, WeChat and Flickr) to keep in touch with peers and tutors?	8.7	7.6	5.4	14.1	**64.1**	10.06	2.96	9.47	21.89	55.62
How often do you use social networking sites (i.e., Facebook, YouTube, Twitter, LinkedIn, and Flickr) to share education-related information?	22.8	13	21.7	21.7	20.7	21.3	10.6	21.9	22.5	**23.7**
How often do you use social networking sites (i.e., Facebook, YouTube, Twitter, LinkedIn, and Flickr) for sharing research, innovations in medicine, and updates in medical field?	30.4	26.1	21.7	9.8	12	**36.1**	22.5	23	8.9	9.5
How often do you read blogs or Wikis for education related information?	12	20.7	37	14.1	**16.3**	14.8	20.1	30.8	20.7	13.6
How often do you contribute to blogs or Wikis to share information, or for dissemination of knowledge?	**84.8**	7.6	4.3	2.2	1.1	82.20	8.30	4.70	3.00	1.80

**Percentages of frequencies of responses to statements about the students’ perceptions of the usage of social networking sites for educational purposes (*N* = 381)**

**Table:**

	Male (n = 137)					Female (n = 244)				
	Strongly agree	Agree	Neutral	Disagree	Strongly disagree	Strongly agree	Agree	Neutral	**Disagree**	**Strongly disagree**
Social networking sites help me in collation of educational materials	26.0	**45.7**	15.2	9.8	3.3	34.3	37.3	17.8	6.5	4.1
Social networking sites are helpful in collaborative and peer-to-peer learning	39.1	37.0	15.2	5.4	3.3	**41.4**	38.5	13.6	5.3	1.2
Social networking sites are useful in developing reading and writing web skills	25.0	**46.7**	13.0	12.0	3.3	32.0	39.0	15.4	11.2	2.4
Social networking sites provide opportunity of virtual meeting with other students and tutors	52.2	38.0	7.6		2.2	**56.2**	35.5	4.7	3.0	0.6
Social networking sites help me to communicate with peers about class projects	53.3	32.6	10.8	2.2	1.1	**59.8**	28.4	5.9	4.1	1.8
Social networking sites help me to access educational resources	39.1	33.7	15.2	8.7	3.3	**56.8**	26.0	10.1	5.3	1.8
Social networking sites help me to retrieve educational references for research	28.3	34.8	10.9	17.4	8.6	**42.6**	21.9	17.2	13.6	4.7
Social networking sites facilitate my professional development of learning skills in technology	28.3	37.0	25.0	6.5	3.2	**38.5**	33.7	17.8	7.7	2.3
Social networking sites are useful in communicating with classmates about course-related topics	**53.3**	32.6	8.7	2.2	3.2	**53.3**	31.4	4.7	7.7	2.9
I have found social networking sites useful during the pre-exam period when I get an instant answer/explanation from my peer, instead of going through the books	**52.2**	29.3	12.0	2.1	4.4	49.1	30.2	9.5	6.5	4.7
I have found social networking sites useful for sharing notes and lectures	**58.7**	26.1	8.7	4.3	2.2	58.0	23.1	10.6	4.7	3.6
I have found social networking sites useful for educational purposes	41.3	35.9	15.2	4.3	3.3	**43.8**	36.6	8.9	6.5	4.2
Medical students need supervision and guidance for the appropriate use of social networking sites for educational purposes	16.3	19.6	23.9	21.7	18.5	21.3	23.1	**26.0**	16.6	13.0
I believe that social networking sites are inappropriate for sharing classroom materials, information, and discussing healthcare related topics	6.5	3.2	19.6	34.8	**35.9**	5.9	6.5	22.2	32.0	33.4

**Percentages of frequencies of responses to statements about the students’ perceptions of the professional use of social networking sites (*N* = 381)**

**Table:**

	**Male (n = 137)**					**Female (n = 244)**				
	Strongly agree	Agree	Neutral	Disagree	Strongly disagree	Strongly agree	Agree	Neutral	Disagree	Strongly disagree
Did you receive formal instruction about the use of SNS in medical school?	6.5	**31.5**	15.2	25.0	21.7	11.2	24.8	17.8	30.8	15.4
Are you familiar with the SNS policy at this institution?	10.8	**34.8**	17.4	26.1	10.9	16.0	29.0	26.0	21.9	7.1
Have you ever closed a personal SNS account for professional reasons?	3.3	12.0	8.6	30.4	**45.7**	5.9	10.1	5.9	37.3	40.8
Do you know the current privacy settings of your personal SNS account?	45.7	33.6	7.6	9.8	3.3	**46.2**	32.0	8.7	9.5	3.6
Do you think that anybody can search you on your SNS account regardless of your privacy settings?	19.6	**35.9**	16.3	18.4	9.8	27.2	35.6	18.9	13.6	4.7
I can permanently delete the content (images/videos/text) I have posted to my SNS account.	18.5	**28.4**	18.5	15.2	19.6	16.0	18.9	17.8	27.8	19.5
I have posted content (images/videos/text) on SNS that could be considered unprofessional.	2.2	14.1	12.0	29.3	42.4	4.1	4.1	6.5	23.7	**61.5**
I have posted content (images/videos/text) on SNS that I now regret.	6.5	10.9	14.1	20.7	47.8	3.0	11.8	5.3	28.4	**51.5**

**The results of the Mann-Whitney U test showing the comparison of the students’ perceptions about the usage of social networking sites among gender (*N* = 381)**

**Table:**

	Male	Female		
	Mean Rank	Mean Rank	Mann-Whitney U	Sig.
How often do you use e-mail for sharing information for educational purpose?	118.46	137.09	6620.50	0.05
How often do you use social networking sites (e.g., Facebook, YouTube, Twitter, LinkedIn, WeChat and Flickr) to keep in touch with peers and tutors?	136.26	127.35	7198.50	0.30
How often do you use social networking sites (i.e., Facebook, YouTube, Twitter, LinkedIn, and Flickr) to share education-related information?	126.22	132.85	7334.00	0.49
How often do you use social networking sites (i.e., Facebook, YouTube, Twitter, LinkedIn, and Flickr) for sharing research, innovations in medicine, and updates in medical field?	135.57	127.72	7261.50	0.40
How often do you read blogs or Wikis for education related information?	130.94	129.48	7595.50	0.88
How often do you contribute to blogs or Wikis to share information, or for dissemination of knowledge?	128.71	131.48	7563.50	0.66
**Educational use of SNS**				
Social networking sites help me in collation of educational materials	136.33	128.10	7284.00	0.37
Social networking sites are helpful in collaborative and peer-to-peer learning	134.76	128.20	7301.50	0.47
Social networking sites are useful in developing reading and writing web skills	135.30	128.66	7378.50	0.47
Social networking sites provide opportunity of virtual meeting with other students and tutors	134.60	129.04	7443.00	0.52
Social networking sites help me to communicate with peers about class projects	136.25	128.14	7291.00	0.35
**Social networking sites help me to access educational resources**	**147.13**	**121.39**	**6198.00**	**0.00**
Social networking sites help me to retrieve educational references for research	140.49	124.32	6689.00	0.08
Social networking sites facilitate my professional development of learning skills in technology	138.96	125.15	6829.00	0.14
Social networking sites are useful in communicating with classmates about course-related topics	128.67	130.72	7523.00	0.82
I have found social networking sites useful during the pre-exam period when I get an instant answer/explanation from my peer, instead of going through the books	126.19	132.07	7297.00	0.51
I have found social networking sites useful for sharing notes and lectures	127.77	130.44	7441.00	0.76
I have found social networking sites useful for educational purposes	132.19	128.04	7354.00	0.65
Medical students need supervision and guidance for the appropriate use of social networking sites for educational purposes	140.23	123.65	6622.00	0.08
I believe that social networking sites are inappropriate for sharing classroom materials, information, and discussing healthcare related topics	133.70	127.21	7216.50	0.48
**Professional use of SNS**				
Did you receive formal instruction about the use of SNS in medical school?	134.46	129.12	7456.00	0.57
Are you familiar with the SNS policy at this institution?	136.77	127.86	7243.00	0.35
Have you ever closed a personal SNS account for professional reasons?	134.02	128.61	7369.50	0.55
Do you know the current privacy settings of your personal SNS account?	130.88	131.07	7762.50	0.98
Do you think that anybody can search you on your SNS account regardless of your privacy settings?	141.92	125.06	6769.50	0.07
I can permanently delete the content (images/videos/text) I have posted to my SNS account.	121.78	136.02	6925.50	0.14
**I have posted content (images/videos/text) on SNS that could be considered unprofessional.**	**113.20**	**140.69**	**6136.00**	**0.00**
I have posted content (images/videos/text) on SNS that I now regret.	124.46	134.56	7172.50	0.26

**The results of the Kruskal Wallis test showing the comparison of the students’ perceptions about the usage of social networking sites across age groups (*N* = 381)**

**Table:**

	18–20	20–23	Above 23		
	Mean Rank	Mean Rank	Mean Rank	Chi-Square	Sig
**How often do you use e-mail for sharing information for educational purpose?**	**117.32**	**144.04**	**151.95**	**10.20**	**0.01**
**How often do you use social networking sites (e.g., Facebook, YouTube, Twitter, LinkedIn, WeChat and Flickr) to keep in touch with peers and tutors?**	**136.09**	**132.67**	**93.08**	**10.58**	**0.01**
**How often do you use social networking sites (i.e., Facebook, YouTube, Twitter, LinkedIn, and Flickr) to share education-related information?**	**139.20**	**127.40**	**92.83**	**10.13**	**0.01**
How often do you use social networking sites (i.e., Facebook, YouTube, Twitter, LinkedIn, and Flickr) for sharing research, innovations in medicine, and updates in medical field?	135.39	125.05	117.80	2.05	0.36
How often do you read blogs or Wikis for education related information?	132.98	125.14	124.73	0.76	0.68
How often do you contribute to blogs or Wikis to share information, or for dissemination of knowledge?	128.72	131.22	132.75	0.25	0.88
**Education use of SNS**					
Social networking sites help me in collation of educational materials	126.40	129.13	154.27	3.84	0.15
Social networking sites are helpful in collaborative and peer-to-peer learning	124.01	134.43	146.90	3.14	0.21
Social networking sites are useful in developing reading and writing web skills	127.50	127.15	154.50	3.86	0.15
Social networking sites provide opportunity of virtual meeting with other students and tutors	121.44	141.44	143.95	6.18	0.05
**Social networking sites help me to communicate with peers about class projects**	**121.12**	**136.89**	**158.25**	**8.88**	**0.01**
Social networking sites help me to access educational resources	125.22	132.24	146.82	2.59	0.28
Social networking sites help me to retrieve educational references for research	127.05	128.29	144.68	1.54	0.46
Social networking sites facilitate my professional development of learning skills in technology	124.25	131.78	148.55	3.04	0.22
Social networking sites are useful in communicating with classmates about course-related topics	125.29	126.55	158.00	6.11	0.05
I have found social networking sites useful during the pre-exam period when I get an instant answer/explanation from my peer, instead of going through the books	123.56	130.44	155.60	5.47	0.07
**I have found social networking sites useful for sharing notes and lectures**	**128.57**	**119.20**	**157.88**	**7.66**	**0.02**
I have found social networking sites useful for educational purposes	124.19	128.87	152.45	4.14	0.13
Medical students need supervision and guidance for the appropriate use of social networking sites for educational purposes	128.64	131.14	124.80	0.18	0.92
I believe that social networking sites are inappropriate for sharing classroom materials, information, and discussing healthcare related topics	136.41	122.31	111.97	4.03	0.13
**Professional use of SNS**					
Did you receive formal instruction about the use of SNS in medical school?	120.75	142.11	145.47	5.97	0.05
Are you familiar with the SNS policy at this institution?	125.37	141.55	124.55	2.85	0.24
Have you ever closed a personal SNS account for professional reasons?	129.30	133.64	123.18	0.52	0.77
Do you know the current privacy settings of your personal SNS account?	130.99	136.90	110.20	3.24	0.20
Do you think that anybody can search you on your SNS account regardless of your privacy settings?	133.46	126.00	128.68	0.59	0.75
**I can permanently delete the content (images/videos/text) I have posted to my SNS account.**	**118.72**	**135.45**	**174.00**	**14.60**	**0.00**
I have posted content (images/videos/text) on SNS that could be considered unprofessional.	129.02	129.68	140.00	0.67	0.72
I have posted content (images/videos/text) on SNS that I now regret.	136.94	120.15	128.13	3.15	0.21

**The results of the Kruskal Wallis test showing the comparison of the students’ perceptions about the usage of social networking sites for educational purposes across years of schooling (*N* = 381)**

**Table:**

		F	1	2	3	4	5		
		Mean Rank	Mean Rank	Mean Rank	Mean Rank	Mean Rank	Mean Rank	Chi-Square	Sig
	**Extent of usage of SNS**								
How often do you use e-mail for sharing information for educational purpose?		105.20	116.92	107.11	99.81	128.45	121.92	5.08	0.28
How often do you use social networking sites (e.g., Facebook, YouTube, Twitter, LinkedIn, WeChat and Flickr) to keep in touch with peers and tutors?		104.76	118.89	116.74	114.58	109.80	102.76	2.08	0.72
How often do you use social networking sites (i.e., Facebook, YouTube, Twitter, LinkedIn, and Flickr) to share education-related information?		102.34	115.36	123.77	116.24	99.50	102.12	4.57	0.33
How often do you use social networking sites (i.e., Facebook, YouTube, Twitter, LinkedIn, and Flickr) for sharing research, innovations in medicine, and updates in medical field?		98.05	110.52	115.80	113.83	114.16	112.54	0.21	1.00
How often do you read blogs or Wikis for education related information?		103.26	99.33	120.10	129.21	109.94	104.36	6.60	0.16
How often do you contribute to blogs or Wikis to share information, or for dissemination of knowledge?		101.36	113.79	114.63	108.38	112.16	117.68	1.05	0.90
	**Educational use of SNS**								
Social networking sites help me in collation of educational materials		97.90	100.52	115.91	125.91	117.34	113.42	4.02	0.40
Social networking sites are helpful in collaborative and peer-to-peer learning		102.78	103.31	114.79	120.16	113.81	117.77	2.09	0.72
**Social networking sites are useful in developing reading and writing web skills**		**95.29**	**91.17**	**132.10**	**119.29**	**120.13**	**102.49**	**14.43**	**0.01**
Social networking sites provide opportunity of virtual meeting with other students and tutors		102.80	102.37	115.67	110.55	118.66	126.42	4.19	0.38
Social networking sites help me to communicate with peers about class projects		97.92	103.49	110.21	117.30	115.22	130.18	5.12	0.28
Social networking sites help me to access educational resources		100.25	98.83	116.17	119.04	117.21	119.70	4.03	0.40
Social networking sites help me to retrieve educational references for research		103.37	99.10	108.40	126.91	124.80	117.92	6.18	0.19
Social networking sites facilitate my professional development of learning skills in technology		98.29	93.91	118.18	127.20	120.25	111.68	7.80	0.10
Social networking sites are useful in communicating with classmates about course-related topics		111.45	114.07	114.95	103.28	111.48	122.71	2.22	0.70
I have found social networking sites useful during the pre-exam period when I get an instant answer/explanation from my peer, instead of going through the books		106.91	106.86	109.02	117.50	111.81	127.28	3.17	0.53
I have found social networking sites useful for sharing notes and lectures		103.76	112.23	116.18	104.34	118.78	115.83	1.49	0.83
I have found social networking sites useful for educational purposes		98.30	107.52	116.63	106.05	120.52	115.22	1.72	0.79
**Medical students need supervision and guidance for the appropriate use of social networking sites for educational purposes**		**105.80**	**92.05**	**116.34**	**132.94**	**123.50**	**105.66**	**11.02**	**0.03**
I believe that social networking sites are inappropriate for sharing classroom materials, information, and discussing healthcare related topics		112.80	117.28	110.62	124.80	107.70	103.30	2.95	0.57
	**Professional use of SNS**								
Did you receive formal instruction about the use of SNS in medical school?		107.89	99.36	105.26	121.16	126.73	130.57	8.24	0.08
Are you familiar with the SNS policy at this institution?		108.03	113.63	104.38	112.54	112.92	133.66	5.16	0.27
Have you ever closed a personal SNS account for professional reasons?		99.49	101.60	107.62	130.16	130.33	107.83	8.19	0.09
**Do you know the current privacy settings of your personal SNS account?**		**97.84**	**109.93**	**119.02**	**93.84**	**103.66**	**140.68**	**13.09**	**0.01**
Do you think that anybody can search you on your SNS account regardless of your privacy settings?		112.87	101.29	125.05	105.48	115.44	119.63	5.09	0.28
I can permanently delete the content (images/videos/text) I have posted to my SNS account.		101.73	105.12	117.58	122.13	120.98	105.26	2.90	0.58
I have posted content (images/videos/text) on SNS that could be considered unprofessional.		104.74	107.11	116.85	114.21	120.08	112.96	1.19	0.88
I have posted content (images/videos/text) on SNS that I now regret.		110.80	117.62	111.96	117.33	121.28	103.05	2.10	0.72

## Appendix 5. A descriptive analysis of the themes, categories and the degree of consensus in rounds 2 and 3 of Delphi study

**Table:**

		Round 2						Round 3						
**Theme: 1 Conformity; self-restraint and subordination of one’s own inclinations to the expectations of others**														
**Category**		**Mode/median**	**Mean/standard deviation (SD)**		**Strong consensus %**			**Mode/median**			**Mean/standard deviation (SD)**		**Strong consensus % (Agree/disagree)**	
**Possesses self-control and self-respect, integrity and honest**		**5.0**	**4.8 ± 0.46**		**100% agreement** **(75% strongly Agree and 25% agree)**			**5.0**			**4.9 ± 0.35**		**100% agreement** **(100% strongly Agree)**	
**Conforms and adheres to the medical bodies’ ethical codes, rules and regulations**		**5.0**	**4.6 ± 0.52**		**100% agreement** **(57% strongly Agree and 43% agree)**			**5.0**			**4.9 ± 0.35**		**100% agreement** **(87.5% strongly Agree and 12.5% agree)**	
**Theme 2: Benevolence: preserve and enhance welfare of those with whom one is in frequent personal contact**														
**Maintains anonymity and confidentiality of others actions**		**5.0**	**4.6 ± 0.52**		**100% agreement** **(62.5% strongly Agree and 37.5% agree)**			**5.0**			**4.8 ± 0.46**		**100% agreement** **(71% strongly Agree and 29% agree)**	
**Says what is for the best of the community, even if it is against his interests**		**5.0**	**4.5 ± 0.93**		**100% agreement** **(71% strongly Agree and 29% agree)**			**5.0**			**4.9 ± 0.35**		**100% agreement** **(87.5% strongly Agree and 12.5% agree)**	
**Dedicated, committed, compassionate, empathetic, helpful and thoughtful**		**5.0**	**4.6 ± 0.52**		**100% agreement** **(57% strongly Agree and 43% agree)**			**5.0**			**4.9 ± 0.35**		**100% agreement** **(87.5% strongly Agree and 12.5% agree)**	
**Has a genuine interest in others and strives to help and elevate others**		**4.5**	**4.4 ± 0.74**		**86% agreement** **(43% strongly Agree and 43% agree)**			**5.0**			**4.8 ± 0.46**		**100% agreement** **(75% strongly Agree and 25% agree)**	
**Theme 3: Power: status and prestige, control people and resources**														
**Confident, advocates patients right, judicial and fair use of power and authority**		**4.5**	**4.4 ± 0.74**		**86% agreement** **(43% strongly Agree and 43% agree)**			**5.0**			**4.9 ± 0.35**		**100% agreement** **(87.5% strongly Agree and 12.5% agree)**	
**Discourages nepotism, religionism, sexism and ageism**		**4.5**	**4.5 ± 0.93**		**100% agreement** **(71% strongly Agree and 29% agree)**			**4.5**			**4.9 ± 0.35**		**100% agreement** **(87.5% strongly Agree and 12.5% agree)**	
**Theme 4: Self-direction: autonomous thought and action (idea of agency)**														
**Praxis, straightforward, treat others as he would like to be treated**		**4.5**	**4.5 ± 0.53**		**100% agreement** **(57% strongly Agree and 43% agree)**			**4.5**			**4.5 ± 0.53**		**100% agreement** **(50% strongly Agree and 50% agree)**	
**Original, flexible, compliant, and pragmatic**		**5.0**	**4.9 ± 0.35**		**100% agreement** **(86% strongly Agree and 14% agree)**			**5.0**			**5.0 ± 0.00**		**100% agreement** **(100% strongly Agree)**	
**Positive mindset with an anticipatory attitude, identifies own deficiencies**		**5.0**	**4.7 ± 0.46**		**100% agreement** **(71% strongly Agree and 29% agree)**			**5.0**			**5.0 ± 0.00**		**100% agreement** **(100% strongly Agree)**	
**Theme 5: Universalism: tolerance and concern for welfare of all others**														
**Advocate of self and other rights thoughtful and helpful**		**4.0**	**4.0 ± 1.07**		**100% agreement** **(50% strongly Agree and 50% agree)**			**4.5**			**4.5 ± 0.53**		**100% agreement** **(43% strongly Agree and 57% agree)**	
**Understands diversity, believes in equality and human rights**		**5.0**	**4.7 ± 0.46**		**100% agreement** **(71% strongly Agree and 29% agree)**			**5.0**			**5.0 ± 0.00**		**100% agreement** **(100% strongly Agree)**	
**Theme 6: Achievement: competitive personal success**														
**Competent, knowledgeable and digitally literate**		**4.0**	**4.2 ± 0.71**		**86% agreement** **(29% strongly Agree and 57% agree)**			**5.0**			**4.8 ± 0.46**		**100% agreement** **(75% strongly Agree and 25% agree)**	
**Theme 7: Honest**														
**Category**	**Mode/median**			**Mean/standard deviation (SD)**		**Strong consensus % (Agree/disagree)**				**Mode/median**		**Mean/standard deviation (SD)**		**Strong consensus % (Agree/disagree)**
**High moral standards and acts with integrity considering security and privacy issues**	**4.5**			**4.5 ± 0.53**		**100% agreement** **(43% strongly Agree and 57% agree)**				**5.0**		**4.3 ± 0.52**		**100% agreement** **(62.5% strongly Agree and 37.5% agree)**
**Maintain relevant security and patient confidentiality in the digital world while remaining accurate**	**5.0**			**4.6 ± 0.52**		**100% agreement** **(57% strongly Agree and 43% agree)**				**5.0**		**4.9 ± 0.35**		**100% agreement** **(87.5% strongly Agree and 12.5% agree)**
**Theme 8: Responsible**														
**Non racial, reliable, tolerant, respects beliefs and cultures of others (also respecting conflicting opinions) in the digital world**		**5.0**		**4.6 ± 0.52**		**100% agreement** **(57% strongly Agree and 43% agree)**	**5.0**					**5.0 ± 0.00**		**100% agreement** **(100% strongly Agree)**
**Considers the privacy issues of others before sharing the information and maintains confidentiality, shares information responsibly**		**5.0**		**4.7 ± 0.46**		**100% agreement** **(71% strongly Agree and 29% agree)**	**5.0**					**4.9 ± 0.35**		**100% agreement** **(87.5% strongly Agree and 12.5% agree)**
**Does not engage in character attacks in digital world**		**5.0**		**4.6 ± 0.74**		**85% agreement** **(71% strongly Agree and 14% agree)**	**5.0**					**4.9 ± 0.35**		**100% agreement** **(87.5% strongly Agree and 12.5% agree)**
**Use appropriate channel (to whistle blow/complain/challenging policy), responsible for own actions or writings**		**4.5**		**4.2 ± 1.03**		**86% agreement** **(43% strongly Agree and 43% agree)**	**5.0**					**4.8 ± 0.46**		**100% agreement** **(75% strongly Agree and 25% agree)**
**Can build therapeutic relationship while maintaining the boundary expected of professionals**		**5.0**		**4.4 ± 1.06**		**86% agreement** **(57% strongly Agree and 29% agree)**	**5.0**					**5.0 ± 0.00**		**100% agreement** **(100% strongly Agree)**
**Theme 9: Self-aware**														
**Accepts feedback and criticism, shows good understanding of self and own’s emotions**	**4.5**			**4.1 ± 1.36**		**86% agreement** **(43% strongly Agree and 43% agree)**			**5.0**			**5.0 ± 0.00**		**100% agreement** **(100% strongly Agree)**
**Up-to-date knowledge, expertise, competent and self-conscious**	**4.5**			**4.1 ± 1.12**		**72% agreement** **(43% strongly Agree and 29% agree)**			**5.0**			**4.8 ± 0.46**		**100% agreement** **(75% strongly Agree and 25% agree)**
**Theme 10: Reflective**														
**Continuous reflection and self-improvement**	**4.5**			**4.0 ± 1.41**		**75% agreement** **(50% strongly Agree and 25% agree)**				**5.0**		**4.6 ± 0.52**		**100% agreement** **(57% strongly Agree and 43% agree)**
**Accountable to self and community**	**4.0**			**3.4 ± 1.60**		**62.5% agreement** **(25% strongly Agree and 37.5% agree)**				**5.0**		**4.2 ± 1.39**		**86% agreement** **(57% strongly Agree and 29% agree)**
**Theme 11: Conscientious**														
**Remains calm and self regulated in case of adversity**	**4.5**			**4.4 ± 0.74**		**86% agreement** **(43% strongly Agree and 43% agree)**				**4.0**		**4.4 ± 0.52**		**100% agreement** **(37.5% strongly Agree and 62.5% agree)**
**Job and institution ethics awareness**	**5.0**			**4.6 ± 0.52**		**100% agreement** **(71% strongly Agree and 29% agree)**				**5.0**		**5.0 ± 0.00**		**100% agreement** **(100% strongly Agree)**
**Aware of consequences of his/her sharing**	**4.5**			**4.2 ± 1.03**		**86% agreement** **(43% strongly Agree and 43% agree)**				**5.0**		**4.8 ± 0.71**		**100% agreement** **(87.5% strongly Agree and 12.5% agree)**
**Committed and motivated for self and others improvement**	**5.0**			**4.9 ± 0.35**		**100% agreement** **(86% strongly Agree and 14% agree)**				**5.0**		**4.6 ± 0.52**		**100% agreement** **(62.5% strongly Agree and 37.5% agree)**
**Able to address infobesity by selecting the right and relevant resources with desired level of evidence**	**5.0**			**4.5 ± 0.76**		**86% agreement** **(57% strongly Agree and 29% agree)**				**5.0**		**4.9 ± 0.35**		**100% agreement** **(87.5% strongly Agree and 12.5% agree)**
**Recognizes boundaries and limitations**	**4.5**			**4.5 ± 0.53**		**100% agreement** **(43% strongly Agree and 57% agree)**				**5.0**		**4.9 ± 0.35**		**100% agreement** **(87.5% strongly Agree and 12.5% agree)**
**Theme 12: Altruistic**														
**Supports and advocates patients rights**	**5.0**			**4.6 ± 0.53**		**86% agreement** **(43% strongly Agree and 43% agree)**				**5.0**		**4.8 ± 0.46**		**100% agreement** **(75% strongly Agree and 25% agree)**
**Caring, sensitive, thoughtful and portrays a persona of caring for others**	**5.0**			**4.5 ± 0.76**		**86% agreement** **(57% strongly Agree and 29% agree)**				**5.0**		**5.0 ± 0.00**		**100% agreement** **(100% strongly Agree)**
**Empowers patients, team members and colleagues**	**5.0**			**4.5 ± 0.76**		**86% agreement** **(57% strongly Agree and 29% agree)**				**5.0**		**4.9 ± 0.35**		**100% agreement** **(87.5% strongly Agree and 12.5% agree)**
**Great regard of profession and clients and fellows**	**5.0**			**4.5 ± 0.76**		**86% agreement** **(57% strongly Agree and 29% agree)**				**5.0**		**4.9 ± 0.35**		**100% agreement** **(87.5% strongly Agree and 12.5% agree)**
**Theme 13: Communicator**														
**Challenges unprofessional behaviors while maintaining professional and good interpersonal skills**	**5.0**			**4.7 ± 0.46**		**100% agreement** **(71% strongly Agree and 29% agree)**			**5.0**			**5.0 ± 0.00**		**100% agreement** **(100% strongly Agree)**
**Brave, distinguished and a good communicator**	**5.0**			**4.4 ± 1.41**		**100% agreement** **(86% strongly Agree and 14% agree)**			**5.0**			**5.0 ± 0.00**		**100% agreement** **(100% strongly Agree)**
**Proactively manages conflict in the online world**	**4.5**			**4.0 ± 1.41**		**72% agreement** **(43% strongly Agree and 29% agree)**			**5.0**			**4.9 ± 0.35**		**100% agreement** **(87.5% strongly Agree and 12.5% agree)**
**Who adheres to professional conduct and shows thoughtfulness towards privileged and accurate communication**	**4.5**			**4.2 ± 1.03**		**86% agreement** **(43% strongly Agree and 43% agree)**			**5.0**			**4.8 ± 0.46**		**100% agreement** **(75% strongly Agree and 25% agree)**

## Appendix 6. Examples of Quotes from Scoping review SNSME students and Delphi Expert Panellists

**Table:**

Framework levels		SNSME Students	Experts
**Mission**			[–By constantly adhering to values mentioned above and reminding about these virtues at early years of life will definitely help to practice it and then own an identity based on mutual respect, safeguarding the rights of others.] [–An individual who understands the nature of online world. Its not contained by boundaries and borders. Anything can go viral. Yet it’s the interface we will be using and breathing in. so the user needs to be sensitive to cultures, individuals, societies, need of institution he/she belongs. Who knows how to avoid racist, cultural and sexist comments? Digitally literate in terms of nations, nationalities and underprivileged. Understands what goes live and what stays behind. [–A growth in terms of professional aspect which will motivate others rather demotivates,,, the deceptive portrayal has made this generation very vulnerable to others achievements/privileges.] [–The real achievements, I mean achievements should shared which add to their professional development and not the luxuries they enjoy broadcasted.] [–In online communication, uplifts colleagues, empowers patients and doesn’t use any information to make them more vulnerable while display and broadcast information which is correct, accurate and helpful for the patients so they are benefitted and the feel supported.]
**Identity**	‘Confidentiality and privacy are fundamental expectations of the patient‒physician relationship.’ Manish Garg 2016 “The majority of nursing school deans surveyed were aware of students posting unprofessional content online and at a higher rate than found in studies conducted among medical and pharmacy schools. This suggests that respondents’ greatest concerns are not with students’ immaturity, but rather with students’ professional identities, including patient confidentiality and civility.” Suzanne Marnocha,2015	[–Covid has brought all interactions and activities to the world where tech savvy generation may have access to everything while no oe is watching. Honest, responsible and thorough people are needed] [–It is difficult to separate digital life from the contexts in which new generation navigate key developmental tasks. The process of acquiring identity is complicated.]	[–Mostly professional identity in digital world is blurred as there is only a thin line that can hardly demarcate professional and personal life. Professional development and digital literary can help develop professional identity in the digital world.] [–Does not spread information that may harm others and doesn’t stay behind false identities as nothing is hidden in this online world and can be perpetually traced back due to archives somewhere all the time.] [–A SNS user that considers the privacy issues of others before sharing the information and the consequences of his/her sharing.] [– Considering curiosity PI shall be acquired and created through the use of innovative online ‘forums’ and ‘networking’ resources resulting in internet-based social interaction for teaching and learning. Whereas an idea and optimum ‘balance’ would require to be maintained between real life and the internet world].
**Values**	‘students believed that maintaining a professional image should not be context specific, many respondents felt that they should not be held to higher standards than the general public’ Jennifer M. Walton 2015 “In the area of information security, prevention always has a lower cost than correction.” DIEGO ADA˜ O FANTI SILVA 2018		[–Applying the rules that he says to himself first and using them. Knowing that he talks to all ages and all types of people at the same time, he needs to adjust what he says to accommodate for that]
**Competencies**			[–In the face of an age of technological transition without precedent, although we are still unaware of the present and or needed Virtues, yet robots, artificial intelligence, the ‘Internet of Things’ and the many other technological advances on the horizon are going to have a lasting impact.]
**Behaviors and environment**	‘healthcare professionals not contact patients through Facebook. Both recommend using high privacy settings and becoming familiar with the terms of use on SNS platforms’ Maude Laliberte 2016 ‘Students feel they can “switch off” their professional identity outside the clinical environment and studies have that shown that students can become detached and “disinhibited when they go online” because of a sense of anonymity.’ P. Kenny 2016 “collaboration with peers and colleagues, SNS provides a platform for surgical or medical education Facilitates more efficient communication with and SNS as an effective marketing or advertising tool when used professionally” Xingbo Long 2017	[–A personal rating will be assigned to each digital user which is linked with the complaints against him/her. Each SNS site implements it.] Who challenges unprofessional behavior while maintaining professional and good interpersonal skills	[–Professional behaviors in digital world are largely influenced by a person’s education, training and interpersonal relations.] [–Understands what right and wrong and abides by the rules while no one is watching, knows that any lapse will be recorded and archived which can be later retrieved.] [–Who treat others with respect, does not engage in character attacks and portrays a persona of caring for others while not compromising his morals and values own morals Who adheres to professional conduct and shows thoughtfulness towards privileged and accurate communication] [– A moderator, pacifier and intelligent individual who know how to diffuse the tension while conveying the message]

## References

[1] Cain J, Romanelli F. E-professionalism: a new paradigm for a digital age. Curr Pharm Teach Learn. 2009;1(2):66–25.

[2] Ellaway RH, Coral J, Topps D, et al. Exploring digital professionalism. Med Teach. 2015;37(9):844–849. 26030375 10.3109/0142159X.2015.1044956

[3] Guraya SY. The usage of social networking sites by medical students for educational purposes: a meta-analysis and systematic review. N Am J Med Sci. 2016;8(7):268. 27583234 10.4103/1947-2714.187131 PMC4982355

[4] Desai SP, Lele V. Correlating internet, social networks and workplace-a case of generation Z students. J Comm Manage Thought. 2017;8(4):802.

[5] Goldie JGS. Connectivism: a knowledge learning theory for the digital age? Med Teach. 2016;38(10):1064–1069. 27128290 10.3109/0142159X.2016.1173661

[6] Fenwick T, Edwards R. Exploring the impact of digital technologies on professional responsibilities and education. Europ Educat Res J. 2016;15(1):117–131.

[7] Zimba O, Radchenko O, Strilchuk L. Social media for research, education and practice in rheumatology. Rheumatol Int. 2020;40(2):183–190. 31863133 10.1007/s00296-019-04493-4

[8] Alhusseini N, Banta JE, Oh J, et al. Social Media Use for Health Purposes by Chronic Disease Patients in the USA. Saudi J Med Med Sci. 2021;9(1):51. 33519344 10.4103/sjmms.sjmms_262_20 PMC7839572

[9] Hill SS, Dore FJ, Steven TE, et al. Twitter use among departments of surgery with general surgery residency programs. J Surg Educ. 2021;78(1):35–42. 32631768 10.1016/j.jsurg.2020.06.008

[10] Alleje ML, Austria BC, Shrestha PA. Social media etiquette in medicine. Br J Hosp Med. 2019;80(9):130–132. 10.12968/hmed.2019.80.9.C130 31498673

[11] Luan H, Wang M, Sokol RL, et al. A scoping review of WeChat to facilitate professional healthcare education in Mainland China. Med Educ Online. 2020;25(1):1782594. 32573367 10.1080/10872981.2020.1782594 PMC7482650

[12] Zeng F, Deng G, Wang Z, et al. WeChat: a new clinical teaching tool for problem-based learning. Int J Med Educ. 2016;7:119. 27111920 10.5116/ijme.5708.e5c4 PMC4844534

[13] Eghtesadi M, Florea A. Facebook, Instagram, Reddit and TikTok: a proposal for health authorities to integrate popular social media platforms in contingency planning amid a global pandemic outbreak. Can J Public Health. 2020;111:389–391. 32519085 10.17269/s41997-020-00343-0 PMC7282468

[14] Bour C, Schmitz S, Ahne A, et al. Scoping review protocol on the use of social media for health research purposes. BMJ Open. 2021;11(2):e040671. 10.1136/bmjopen-2020-040671 PMC7880087 33574143

[15] Singh T, Roberts K, Cohen T, et al. Social Media as a Research Tool (SMaaRT) for risky behavior analytics: methodological review. JMIR Public Health Surveill. 2020;6(4):e21660. 33252345 10.2196/21660 PMC7735906

[16] Spector ND, Matz PS, Levine LJ, et al. e-Professionalism: challenges in the age of information. J Pediatr. 2010;156(3):345–346. 20176180 10.1016/j.jpeds.2009.12.047

[17] Seidel RL, Jalilvand A, Kunjummen J, et al. Radiologists and social media: do not forget about Facebook. J Am College Radiol. 2018;15(1):224–228. 10.1016/j.jacr.2017.09.013 29132999

[18] Patel MS, Davis MM, Lypson ML. Advancing medical education by teaching health policy. N Engl J Med. 2011;364(8):695–697. 21345098 10.1056/NEJMp1009202

[19] Monrouxe LV. Identity, identification and medical education: why should we care? Med Educ. 2010;44(1):40–49. 20078755 10.1111/j.1365-2923.2009.03440.x

[20] Gholami-Kordkheili F, Wild V, Strech D. The impact of social media on medical professionalism: a systematic qualitative review of challenges and opportunities. J Med Internet Res. 2013;15(8):e184. 23985172 10.2196/jmir.2708 PMC3758042

[21] Chou W-YS, Oh A, Klein WM. Addressing health-related misinformation on social media. Jama. 2018;320(23):2417–2418. 30428002 10.1001/jama.2018.16865

[22] Peck JL. Social media in nursing education: responsible integration for meaningful use. J Nurs Educ. 2014;53(3):164–169. 24530130 10.3928/01484834-20140219-03

[23] Muls J, Thomas V, De Backer F, et al. Identifying the nature of social media policies in high schools. Educat Inform Technol. 2020;25(1):281–305.

[24] Brody H, Doukas D. Professionalism: a framework to guide medical education. Med Educ. 2014;48(10):980–987. 25200018 10.1111/medu.12520

[25] Billett S. Cooke, M., Irby, DM & O’Brien, BC. Educating Physicians: A Call for Reform of Medical School and Residency. The Carnegie Foundation for the Advancement of Teaching. 10.1080/10401334.2013.842915 24246101

[26] Frank JR, Snell LS, Cate OT, Holmboe ES, Carraccio C, Swing SR, et al. Competency-based medical education: theory to practice. Med Teach. 2010;32(8):638–645. 20662574 10.3109/0142159X.2010.501190

[27] Cruess RL, Cruess SR, Boudreau JD, et al. A schematic representation of the professional identity formation and socialization of medical students and residents: a guide for medical educators. Acad Med. 2015;90(6):718–725. 25785682 10.1097/ACM.0000000000000700

[28] Bilau AA, Witt E, Lill I. Research methodology for the development of a framework for managing post-disaster housing reconstruction. Procedia Eng. 2018;212:598–605.

[29] Corti L. The European landscape of qualitative social research archives: Methodological and practical issues. InForum: Qualitative Social Research 2011 (Vol. 12, No. 3). FQS.

[30] Guraya SY, Almaramhy H, Al-Qahtani MF, et al. Measuring the extent and nature of use of Social Networking Sites in Medical Education (SNSME) by university students: results of a multi-center study. Med Educ Online. 2018;23(1):1505400. 30081773 10.1080/10872981.2018.1505400 PMC6084502

[31] Guraya SY, Al-Qahtani MF, Bilal B, et al. Comparing the extent and pattern of use of social networking sites by medical and non medical university students: a multi-center study. Psychol Res Behav Manag. 2019;12:575. 31534376 10.2147/PRBM.S204389 PMC6681135

[32] Guraya SS, Guraya SY, Yusoff MSB. Preserving professional identities, behaviors, and values in digital professionalism using social networking sites; a systematic review. BMC Med Educ. 2021;21(1):1–12. 34247617 10.1186/s12909-021-02802-9 PMC8273947

[33] Guraya S. Prognostic significance of circulating microRNA-21 expression in esophageal, pancreatic and colorectal cancers; a systematic review and meta-analysis. Int J Surg. 2018;60:41–47. 30336280 10.1016/j.ijsu.2018.10.030

[34] Cooke A, Smith D, Beyond BA. PICO: the SPIDER tool for qualitative evidence synthesis. Qual Health Res. 2012;22(10):1435–1443. 22829486 10.1177/1049732312452938

[35] Morgan MP, Thomas W, Rashid-Doubell F. Academic staff perspectives on delivering a shared undergraduate medical module on three transnational campuses: practical considerations and lessons learned. Med Teach. 2020;42(1):36–38. 31411913 10.1080/0142159X.2019.1649380

[36] Hsu -C-C, Sandford BA. The Delphi technique: making sense of consensus. Pract Assess Res Eval. 2007;12(1):10.

[37] List D. Maximum variation sampling for surveys and consensus groups. Adelaide: audience Dialogue. J Qualitat Res EducatJOQRE. 2004.

[38] Saldana J. Analyzing longitudinal qualitative observational data. Handbook of longitudinal research: design, measurement, and analysis. 2008;297–311.

[39] Watling CJ, Lingard L. Grounded theory in medical education research: AMEE Guide No. 70. Med Teach. 2012;34(10):850–861. 22913519 10.3109/0142159X.2012.704439

[40] Farmer T, Robinson K, Elliott SJ, et al. Developing and implementing a triangulation protocol for qualitative health research. Qual Health Res. 2006;16(3):377–394. 16449687 10.1177/1049732305285708

[41] Foster RL. Addressing epistemologic and practical issues in multimethod research: a procedure for conceptual triangulation. Adv Nurs Sci. 1997;20(2):1–12. 10.1097/00012272-199712000-00002 9398934

[42] Guba EG, Lincoln YS. Competing paradigms in qualitative research. Handbook Qualitat Res. 1994;2(163–194):105.

[43] Yuan B, Li J. The policy effect of the general data protection regulation (GDPR) on the digital public health sector in the european union: an empirical investigation. Int J Environ Res Public Health. 2019;16(6):1070. 10.3390/ijerph16061070 PMC6466053 30934648

[44] Miles MB, Huberman AM, and Saldaña J. Qualitative data analysis: a methods sourcebook (3rd ed.). London, UK: Sage publications; 2014.

[45] Korthagen FA. In search of the essence of a good teacher: towards a more holistic approach in teacher education. Teaching Teacher Educ. 2004;20(1):77–97.

[46] Choudhury S, McKinney KA. Digital media, the developing brain and the interpretive plasticity of neuroplasticity. Transcult Psychiatry. 2013;50(2):192–215. 23599391 10.1177/1363461512474623

[47] Firth J, Torous J, Stubbs B, et al. The “online brain”: how the Internet may be changing our cognition. World Psychiatry. 2019;18(2):119–129. 31059635 10.1002/wps.20617 PMC6502424

[48] Coulehan J. Today’s Professionalism: engaging the Mind but Not the Heart. Acad Med. 2005;80(10):892–898. 16186604 10.1097/00001888-200510000-00004

[49] Schenarts PJ. Now arriving: surgical trainees from generation Z. J Surg Educ. 2020;77(2):246–253. 31562032 10.1016/j.jsurg.2019.09.004

[50] Jha V, Brockbank S, Roberts T. A framework for understanding lapses in professionalism among medical students: applying the theory of planned behavior to fitness to practice cases. Acad Med. 2016;91(12):1622–1627. 27355781 10.1097/ACM.0000000000001287

[51] Crone EA, Konijn EA. Media use and brain development during adolescence. Nat Commun. 2018;9(1):1–10. 29467362 10.1038/s41467-018-03126-x PMC5821838

[52] PrakashYadav G, Rai J. The Generation Z and their social media usage: a review and a research outline. Global J Enterprise Inform Syst. 2017;9(2):110–116.

[53] Holden M, Buck E, Clark M, Szauter K, Trumble J.Professional identity formation in medical education: The convergence of multiple domains. HEC Forum. 2012;24:245–255. 10.1007/s10730-012-9197-6 23104548

[54] Knight LV, Mattick K. ‘When I first came here, I thought medicine was black and white’: making sense of medical students’ ways of knowing. Soc Sci Med. 2006;63(4):1084–1096. 16519978 10.1016/j.socscimed.2006.01.017

[55] Davis JL, Jurgenson N. Context collapse: theorizing context collusions and collisions. Inform Comm Soc. 2014;17(4):476–485.

[56] Marwick AE, Boyd D. I tweet honestly, I tweet passionately: twitter users, context collapse, and the imagined audience. New Med Soc. 2011;13(1):114–133.

[57] Kesselheim JC, Batra M, Belmonte F, et al. New professionalism challenges in medical training: an exploration of social networking. J Grad Med Educ. 2014;6(1):100. 24701318 10.4300/JGME-D-13-00132.1 PMC3963763

[58] Gordon L, Rees CE, Jindal‐Snape D. Doctors’ identity transitions: choosing to occupy a state of ‘betwixt and between’. Med Educ. 2020;54(11):1006–1018. 32402133 10.1111/medu.14219

[59] Bergner RM, Holmes JR. Self-concepts and self-concept change: a status dynamic approach. Psychotherapy. 2000;37(1):36.

[60] Antoniou SA, Antoniou GA, Granderath FA, et al. Reflections of the Hippocratic Oath in modern medicine. World J Surg. 2010;34(12):3075–3079. 20814679 10.1007/s00268-010-0604-3

[61] Kohlberg L, Hersh RH. Moral development: a review of the theory. Theory into Pract. 1977;16(2):53–59.

[62] O’Regan A, Smithson WH, Spain E. Social media and professional identity: pitfalls and potential. Med Teach. 2018;40(2):112–116. 29172814 10.1080/0142159X.2017.1396308

[63] Erikson EH. Eight ages of man. Int J Psychiat. 1966. 5934808

[64] Marcia JE. Development and validation of ego-identity status. J Pers Soc Psychol. 1966;3(5):551. 5939604 10.1037/h0023281

[65] Van Der Zwet J, Zwietering P, Teunissen P, et al. Workplace learning from a socio-cultural perspective: creating developmental space during the general practice clerkship. Adv Health Sci Educ. 2011;16(3):359–373. 10.1007/s10459-010-9268-x PMC3139899 21188514

[66] Ruan B, Yilmaz Y, Lu D, et al. Defining the Digital Self: a Qualitative Study to Explore the Digital Component of Professional Identity in the Health Professions. J Med Internet Res. 2020;22(9):e21416. 32990636 10.2196/21416 PMC7556376

